# mRNA‐based Vaccines Targeting the T‐cell Epitope‐rich Domain of Epstein Barr Virus Latent Proteins Elicit Robust Anti‐Tumor Immunity in Mice

**DOI:** 10.1002/advs.202302116

**Published:** 2023-10-27

**Authors:** Ge‐Xin Zhao, Guo‐Long Bu, Gang‐Feng Liu, Xiang‐Wei Kong, Cong Sun, Zi‐Qian Li, Dan‐Ling Dai, Hai‐Xia Sun, Yin‐Feng Kang, Guo‐Kai Feng, Qian Zhong, Mu‐Sheng Zeng

**Affiliations:** ^1^ State Key Laboratory of Oncology in South China Collaborative Innovation Center for Cancer. Medicine Guangdong Key Laboratory of Nasopharyngeal Carcinoma, Diagnosis, and Therapy Sun Yat‐sen University Cancer Center Guangzhou 510060 China; ^2^ Department of Head and Neck Surgery Section II The Third Affiliated Hospital of Kunming Medical University/Yunnan Cancer Hospital 519 Kunzhou Road Kunming 650118 China; ^3^ Guangdong‐Hong Kong Joint Laboratory for RNA Medicine Sun Yat‐sen University Cancer Center Guangzhou 510060 China

**Keywords:** cancer immunotherapies, Epstein‐Barr virus (EBV), mRNA vaccines, nasopharyngeal carcinoma (NPC)

## Abstract

Epstein‐Barr virus (EBV) is associated with various malignancies and infects >90% of the global population. EBV latent proteins are expressed in numerous EBV‐associated cancers and contribute to carcinogenesis, making them critical therapeutic targets for these cancers. Thus, this study aims to develop mRNA‐based therapeutic vaccines that express the T‐cell‐epitope‐rich domain of truncated latent proteins of EBV, including truncatedlatent membrane protein 2A (Trunc‐LMP2A), truncated EBV nuclear antigen 1 (Trunc‐EBNA1), and Trunc‐EBNA3A. The vaccines effectively activate both cellular and humoral immunity in mice and show promising results in suppressing tumor progression and improving survival time in tumor‐bearing mice. Furthermore, it is observed that the truncated forms of the antigens, Trunc‐LMP2A, Trunc‐EBNA1, and Trunc‐EBNA3A, are more effective than full‐length antigens in activating antigen‐specific immune responses. In summary, the findings demonstrate the effectiveness of mRNA‐based therapeutic vaccines targeting the T‐cell‐epitope‐rich domain of EBV latent proteins and providing new treatment options for EBV‐associated cancers.

## Graphical abstract

mRNA‐based vaccines expressing truncated Epstein‐Barr virus (EBV) latent protein domains effectively elicit cellular and humoral immunity, inhibit tumor progression, and improve the survival of tumor‐bearing mice. Truncated antigens show superior immune activation compared to full‐length antigens, offering promising therapeutic options for EBV‐related malignancies.

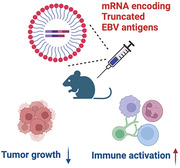

## 1 Introduction

Epstein‐Barr virus (EBV) is the first identified oncogenic virus in humans and has infected more than 90% of the global population^[^ ^1^ ^]^ It mainly infects epithelial cells and B cells and accounts for ≈2% of human malignant tumors.^[^ ^2^, ^3^ ^]^ Since the first report of EBV in Burkitt lymphoma in 1964, EBV has been closely linked to numerous lymphoid and epithelial malignancies, such as Hodgkin lymphoma, Burkitt lymphoma, gastric cancer, nasopharyngeal carcinoma (NPC), and post‐transplant lymphoproliferative disease.^[^ ^4^ ^]^

The persistence of EBV is due to its ability to establish latent infection and evade immune recognition in host cells.^[^ ^5^ ^]^ Generally, EBV infection occurs during childhood and is often asymptomatic.^[^ ^6^, ^7^ ^]^ Subsequently, EBV can establish latent infection and evade immune recognition in the host cells and can typically persist for the lifetime of the host.^[^ ^8^ ^]^ During latency, only a few EBV genes are expressed, including those for EBV nuclear antigens (EBNAs) and latent membrane proteins (LMPs). LMP2A is generally expressed in NPC and can be detected in other EBV‐related malignancies, such as gastric carcinoma and Hodgkin's disease.^[^ ^9^, ^10^ ^]^ LMP2A protein has been identified in ≈50% of NPC biopsies using immunohistochemistry (IHC), and the LMP2A mRNA has been detected in more than 95% of the tumor samples.^[^ ^11^, ^12^ ^]^ In contrast, only three of 18 NPC biopsy specimens have shown a definite mRNA‐specific signal for LMP‐1.^[^ ^12^ ^]^ EBNA1 is persistently expressed in EBV‐related tumors and is crucial for maintaining and replicating the EBV genome, while EBNA3A is upregulated in EBV‐induced lymphoma and can induce potent anti‐EBV‐specific cytotoxic T lymphocytes (CTLs) in vitro.^[^ ^13^, ^14^ ^]^ In addition, compared with the control lymphocytes from healthy humans, the EBV‐induced lymphoma cells exhibit 38‐, 1157‐, and 1154‐fold increase in the mRNA levels of LMP2A, EBNA1, and EBNA3A, respectively.^[^ ^13^, ^15^ ^]^ Therefore, these EBV latent proteins are promising candidates for therapeutic vaccines.

The presence of latent antigens contributes remarkably to the tumorigenic potential of EBV.^[^ ^13^ ^]^ Therefore, it is imperative to implement strategies aimed at minimizing the influence of these antigens when designing a vaccine. Specifically, LMP2A is predominantly located at the invasion frontier and can considerably enhance the invasive and migratory abilities of NPC cells.^[^ ^16^ ^]^ The functional tyrosine activation domain of LMP2A is situated at its N‐terminal region and can recruit tyrosine kinases to activate downstream B‐cell activation pathways.^[^ ^17^ ^]^ EBNA1 is closely associated with epithelial‐mesenchymal transition and mediates growth‐transforming activities by triggering the transcription of latent genes essential for the immortalization of tumor cells.^[^ ^18^ ^]^ Furthermore, EBNA1 promotes the chemotactic migration of regulatory T (Treg) cells in the NPC microenvironment, contributing to cell immortalization and tumorigenesis.^[^ ^19^, ^20^ ^]^ Finally, EBNA3A inhibits multiple tumor suppressor proteins and is critical for maintaining the growth of lymphoblastoid cell lines.^[^ ^21^, ^22^ ^]^ Therefore, the development of an efficient therapeutic vaccine against EBV requires the induction of efficient antitumor immune responses while simultaneously evading the potential transforming capacity of EBV latent proteins. To address this, the present study aimed to design truncated antigens that lack transforming domains yet cover major T‐cell epitopes. This innovative approach was employed to minimize the potential risks associated with the induction of tumorigenicity while still stimulating an effective antitumor‐immune response.

Previous studies have clinically evaluated the efficacy of EBV therapeutic vaccines utilizing dendritic cells (DCs) and recombinant viral vectors.^[^ ^23^, ^24^, ^25^ ^]^ While these vaccines have demonstrated potential clinical benefits, they have not yet been approved for clinical use.^[^ ^23^, ^26^ ^]^ DC‐based EBV vaccines have shown potential in preclinical studies and early‐phase clinical trials, particularly for the treatment of EBV‐associated malignancies.^[^ ^24^, ^27^ ^]^ These vaccines can effectively stimulate specific immune responses against EBV in vaccinated patients.^[^ ^27^ ^]^ However, manufacturing and scalability challenges have hindered their progress toward commercialization.^[^ ^28^ ^]^

mRNA‐based vaccines have recently emerged as a promising technology for cancer vaccine development due to their ability to induce safe, effective, and durable immune responses.^[^ ^29^, ^30^, ^31^ ^]^ These vaccines can efficiently elicit both cellular and humoral immune responses, and they do not produce significant anti‐vector immunity, which allows for repeated administration.^[^ ^32^, ^33^, ^34^, ^35^ ^]^ Moreover, mRNA vaccines do not contain DNA, eliminating the risk of genomic integration. Their favorable safety profile is evident by the impossibility of integration into the host genome or reactivation of latent viruses, as observed in coronavirus disease mRNA vaccines.^[^ ^35^ ^]^ Furthermore, mRNA can be directly expressed in vivo, making it an attractive alternative for antigens that are difficult to purify in vitro.^[^ ^36^ ^]^

## 2 Results

### 2.1 Design and Expression of mRNA Encoding Truncated EBV Antigens with Enriched T Cell Epitopes

**Figure 1:**
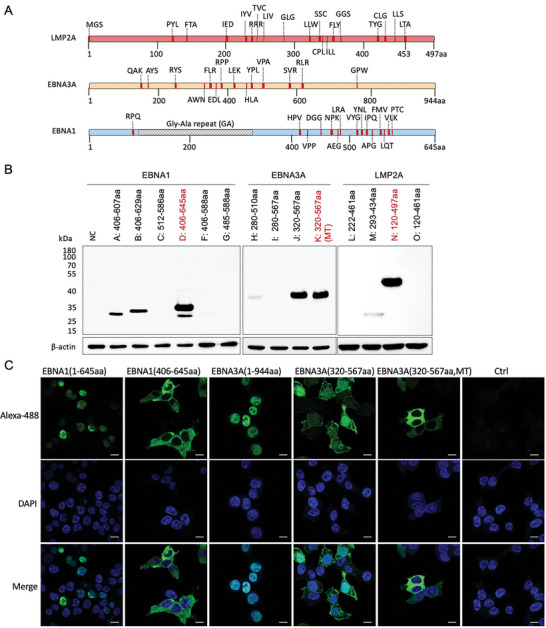
Mapping of T‐cell‐epitopes on Epstein–Barr virus (EBV) latent antigens and expression of latent membrane proteins (LMP)−2A, EBV nuclear antigens (EBNA)−1, and EBNA3A truncations in vitro. A) T‐cell epitope maps of LMP2A, EBNA1, and EBNA3A. Epitopes were identified from previous studies (data source: IDEB.org) and are illustrated as vertical red bars, identified by the first three amino acids. Thick red bars represent epitopes reported by at least three references, while thin red bars represent epitopes identified by two references. Full details of epitopes are given in Table S1 (Supporting Information). B) Western blot analysis for the expression level of flag‐tagged LMP2A, EBNA1, and EBNA3A truncations is presented. After mRNA transfection into 293T cells for 12 h, cell lysates were analyzed using western blotting, with anti‐Flag tag and anti‐β‐actin antibodies used as primary antibodies. The mutated nuclear localization sequence (K378A, R379A, K397A, R398A) of EBNA3A protein is denoted as MT. C) Representative confocal images of 293T cells expressing flag‐tagged EBNA1 and EBNA3A truncations. The corresponding mRNA was transfected into 293T cells 12 h before detection. Protein expression was detected with Alexa Fluor 488 Conjugated anti‐flag antibody(green), and the nuclei were stained with 4′,6‐diamidino‐2‐phenylindole (DAPI) (blue). Scale Bar = 10 µm.

The T cell epitopes are unevenly distributed within EBV latent antigens, including LMP2A, EBNA1, and EBNA3A.^[^ ^37^, ^38^ ^]^ To develop a more potent antitumor immune response, we analyzed previously reported T cell epitopes in these antigens (**Figure** **1**A, Table S1, Supporting Information) and generated truncated variants of EBV antigens that retained the major T cell epitopes.^[^ ^39^, ^40^, ^41^ ^]^

Six, four, and four truncated variants of EBNA1, EBNA3A, and LMP2A, respectively, were designed in this study. To confirm the successful expression of these variants in vitro, 293T cells were transiently transfected with mRNA, and antigen expression was analyzed after 12 h using western blotting. The most effective expressed truncated variants that covered adequate T‐cell epitopes were selected for further evaluation; these included Trunc‐LMP2A (LMP2A 120–461aa), Trunc‐EBNA1 (EBNA1 406–645aa), and Trunc‐EBNA3A (EBNA3A 320–567aa) (Figure 1B; Figure S1A, Supporting Information).

Previous studies have highlighted the tumorigenic functional domains in latent proteins.^[^ ^42^, ^43^ ^]^ LMP2A, for example, is known for its potent transforming ability mediated by its constitutively phosphorylated tyrosine residues at the N‐terminal tail.^[^ ^43^, ^44^ ^]^ EBNA1 and EBNA3A are nuclear antigens that bind DNA or interact with DNA‐binding proteins.^[^ ^45^ ^]^ To minimize the potential effects of EBV antigens on host cells, we introduced specific mutations. For instance, we mutated the nuclear localization sequences (NLS) in Trunc‐EBNA3A to PKVAAPP and RAGAA from the original PKVKRPP and RAGKR sequences, respectively.^[^ ^46^, ^47^ ^]^ Additionally, we excluded the N‐terminal of LMP2A from Trunc‐LMP2A since the three tyrosine residues, Y74, Y85, and Y112, are critical for the LMP2A signal transduction.^[^ ^42^, ^48^ ^]^ Furthermore, to avoid inducing multiple sclerosis, which is associated with B cell responses against EBNA1 386–405aa, we did not include this region in the Trunc‐EBNA1.^[^ ^49^, ^50^ ^]^

To ensure that truncated antigens do not promote proliferation, we transfected 4T1 cells with truncated or full‐length antigens and observed no significant changes in cell proliferation rates (Figure S2A, Supporting Information). We also verified the cellular location of NLS‐mutated Trunc‐EBNA1 and Trunc‐EBNA3A by transfecting mRNA encoding Trunc‐EBNA1, Trunc‐EBNA3A, or their natural form into 293T cells. Fluorescence microscopy revealed that the NLS‐mutated truncations did not enter the cell nucleus, whereas full‐length EBNA1, EBNA3A, and wild‐type truncated EBNA3A were detected in the nuclei (Figure 1C).

### 2.2 Characterization of mRNA‐Liposome Nanoparticle Complex

**Figure 2:**
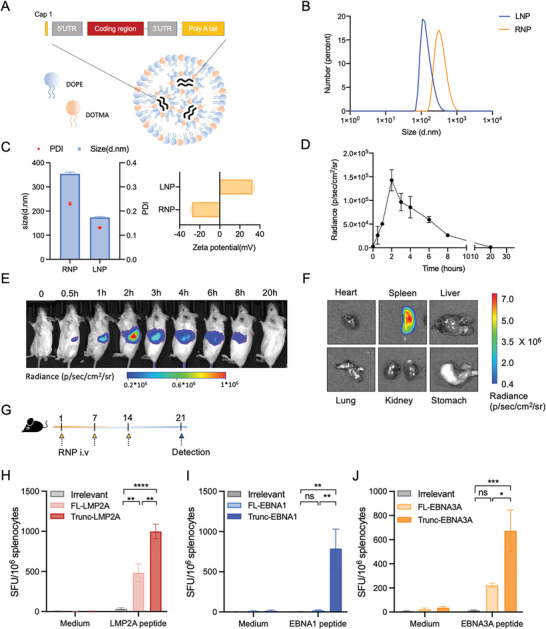
Characterization of mRNA‐liposome nanoparticle (RNP) and cellular immune response elicited by Trunc‐LMP2A‐RNP, Trunc‐EBNA1‐RNP, and Trunc‐EBNA3A‐RNP. A) Schematic representation of the structure and components of the RNP. The liposomes (LNP) were composed of DOTMA (blue) and DOPE (orange). B,C) The particle size (B and C), polydispersity index (PDI) (C, red dots), and zeta potential (C, yellow bars) of the RNP and LNP (*n =* 3). D,E) Bioluminescence imaging of BALB/c mice (*n =* 2) after intravenous (i.v.) injection of 20 µg luciferase RNP. F) Bioluminescence imaging of organs in a BALB/c mouse 2 h after i.v. injection of 20 µg luciferase RNP. G) C57BL/6 mice (*n =* 6) were intravenously immunized with 20 µg RNPs encoding truncated/full‐length EBV latent antigens (LMP2A, EBNA3A, or EBNA1) or irrelevant RNP control (NC) on day 1, 7, and 14. On day 21, immunized mice were euthanized. The spleens and other major organs were collected for determining T‐cell responses and histological analysis. H–J) Frequencies of interferon (IFN)‐γ releasing antigen‐specific cells demonstrated using ELISpot assay. Mice spleens (*n =* 6) were removed on day 21, and 2 × 10^5^ splenocytes were co‐incubated with 10 µg mL^−1^ corresponding peptide pools (LMP2A, EBNA3A, or EBNA1). Phorbol myristate acetate (PMA) plus ionomycin and medium alone served as positive and negative controls, respectively. SFU, spot‐forming units; IFN‐γ, interferon‐gamma. The irrelevant RNP contained mRNA encoding the green fluorescent protein (EGFP). Significance was determined using a one‐way analysis of variance (ANOVA) followed by Tukey's multiple comparisons test (H–J). Error bars, mean ± standard error of the mean (SEM). *p < 0.05; **p < 0.01; ***p < 0.001.

To efficiently generate an in vivo immune response, we applied an mRNA delivery method that specifically targets the spleen, as previously reported.^[^ ^51^ ^]^ We utilized cationic liposomes composed of dioleoylphosphatidylethanolamine (DOPE) and cationic lipid *N*‐[1‐(2,3‐dioleyloxy)propyl]‐*N*, *N*, *N*‐trimethylammonium chloride (DOTMA), which can form stable nanoparticles with mRNA and have an encapsulation efficacy of >90% (**Figure** **2**A, Figure S2B,C, Supporting Information). Liposomes were initially prepared at a particle size of 180 nm and then packaged with mRNA, thus increasing the size of the mRNA‐liposome nanoparticle complex (RNP) particles to ≈340 nm (Figure 2B,C), and the ionizable lipid nitrogen and oligonucleotide phosphate (N/P) ratio used in the study was 2:1.3 (Figure S2D, Supporting Information). The zeta potential of liposomes alone was ≈ +30 mV, whereas that of the RNPs shifted to ≈ −30 mV (Figure 2C). This negatively charged mRNA delivery system could specifically target the spleen when injected intravenously.^[^ ^51^ ^]^ To verify this, we packaged mRNA encoding luciferase into RNP (luciferase‐RNP) and injected it intravenously into mice to assess the expression profile of luciferase signaling. The results demonstrated that the luciferase signal peaked at ≈2 h (Figure 2D) and was predominantly expressed in the spleen (Figure 2E,F). We also investigated the stability of the mRNA and liposomes in vitro. The purified mRNA did not degrade significantly for at least 3 days at 37 °C and remained stable for at least six months at −20 °C (Figure S3A, Supporting Information). Additionally, the size and zeta potential of the liposomes were unchanged for up to 12 months at 4 °C, while those of the RNPs remained stable at 4 °C for up to 2 days (Figure S3B–E, Supporting Information). Furthermore, even after 12 months of storage, the liposomes could form stable RNPs and exhibited potent expression, similar to freshly generated liposomes (Figure S3F, Supporting Information).

### 2.3 Robust Immune Responses in Mice Elicited by RNPs Containing Truncated EBV Antigens

The efficacies of RNPs encoding Trunc‐LMP2A, Trunc‐EBNA1, and Trunc‐EBNA3A in inducing an immune response were assessed in C57BL/6 mice (Figure 2G). The mice were grouped and received intravenous administration of 20 µg RNPs encoding the respective antigens or irrelevant controls on days 1, 7, and 14. On day 21, the interferon‐γ (IFN‐γ) response was measured using ELISpot after extracting the spleens from the mice (Figure 2G). Antigen‐specific IFN‐γ responses were detected in all vaccinated groups except for mice vaccinated with full length‐EBNA1‐RNP (FL‐EBNA1‐RNP) or irrelevant‐RNP (Figure 2H–J; Figures S4A and 3C, Supporting Information). Surprisingly, Trunc‐LMP2A‐RNPs elicited a robust IFN‐γ response at higher levels than those by FL‐LMP2A‐RNPs (Figure 2H, Figure S4A, Supporting Information). Moreover, the total cellular response against EBNA3A peptides in mice treated with Trunc‐EBNA3A‐RNP was 674 ± 172 spot‐forming units (SFU)/10^6^ splenocytes, which was significantly greater than that observed in mice treated with FL‐EBNA3A‐RNP (221 ± 18 SFU/10^6^ splenocytes) (Figure 2J, Figure S4C, Supporting Information).

To investigate the immune activation effect of RNP in vivo, we assessed the activation of T cells, natural killer (NK) cells, and the maturation of DCs 24 h after administration (Figure S5A–D, Supporting Information). We found that both the irrelevant RNP and Trunc‐LMP2A RNP strongly induced the activation of NK cells, CD4+, and CD8+ T cells, as evidenced by upregulation of the activation marker, CD69 (Figure S5A,C, Supporting Information). Additionally, the maturation of DCs in the spleens of mice was observed, characterized by the upregulation of activation markers CD40 and CD86 (Figure S5B,D, Supporting Information). Furthermore, we observed a significant upregulation of cytokines such as IL‐6, IFN‐α, and TNF‐α in peripheral blood at the mRNA level six hours after RNP injection, indicating the activation of innate immune response.^[^ ^51^, ^52^ ^]^ (Figure S5E, Supporting Information).

To assess the safety of RNP vaccines, we conducted biochemical assays and examined histopathological changes in vital organs (heart, kidney, liver, and lung) of vaccinated mice (Figure S6A,B, Supporting Information). The results showed no significant changes in alanine transaminase (ALT), aspartate aminotransferase (AST), total protein (TP), UREA, and uric acid (UA) levels between the control and RNP‐treated groups (Figure S6A, Supporting Information); no pathological changes were observed in the vaccinated mice compared to those in untreated mice (Figure S6B, Supporting Information). Additionally, there was no significant difference in body weight observed between the two groups (Figure S6D, Supporting Information).

### 2.4 Superior Protection Provided by Trunc‐LMP2A‐RNP Compared to FL‐LMP2A‐RNP in Mice with Tumors

**Figure 3:**
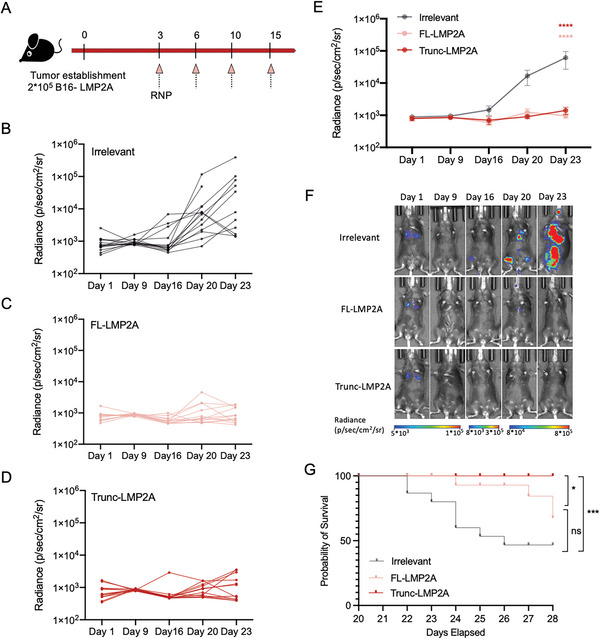
Anti‐tumor activities of Trunc‐LMP2A‐RNP and FL‐LMP2A‐RNP. (A) C57BL/6 mice were injected intravenously with B16‐LMP2A cells (2 × 10^5^ per mouse). Mice were randomly divided into three groups and immunized with 40 µg Trunc‐LMP2A‐RNP (*n =* 12), FL‐LMP2A‐RNP (*n =* 12), or irrelevant RNP (*n =* 14) on days 3, 6, 10, and 15 via intravenous injection. B–F) In vivo bioluminescence imaging of tumor growth. B–D) Individual tumor growth curves, E) average bioluminescent signals, and F) representative in vivo bioluminescence images of mice from the three groups. G) Kaplan–Meier survival curves for tumor‐bearing mice treated with Trunc‐LMP2A‐RNP, FL‐LMP2A‐RNP, or irrelevant RNP. Significance was determined using two‐way ANOVA followed by Dunnett's multiple comparisons test (E) or log‐rank test (G). Error bars, mean ± SEM. *p < 0.05; **p < 0.01; ***p < 0.001; ****p < 0.0001.

We further investigated whether the improved antitumor effects of Trunc‐LMP2A‐RNP correlated with stronger T‐cell immunity. We first inoculated C57BL/6 mice with 2 × 10^5^ B16 cells expressing full‐length LMP2A and then administered RNP vaccines via intravenous injections (**Figure** **3**A). Compared to the irrelevant‐RNP control, Trunc‐LMP2A‐RNP and FL‐LMP2A‐RNP exhibited comparable inhibitory effects on tumor progression based on the bioluminescence signal by day 23 (Figure 3B–F). However, mice treated with Trunc‐LMP2A‐RNP had significantly longer survival times than those of mice treated with FL‐LMP2A‐RNP (Figure 3G). This indicates that Trunc‐LMP2A‐RNP provided better protection in tumor‐bearing mice than that by FL‐LMP2A‐RNP. Further, we used a subcutaneous tumor model in which 1× 10^5^ 4T1 cells overexpressing LMP2A were inoculated subcutaneously into BALB/c mice (Figure S7A, Supporting Information). The inhibition of tumor progression by Trunc‐LMP2A‐RNP and FL‐LMP2A‐RNP was significantly more potent than that in the irrelevant‐RNP group (Figure S7B, Supporting Information). On day 28, ≈85% (6/7) of the mice in the irrelevant‐RNP group had died. While one mouse (1/6) in the FL‐LMP2A‐RNP group died on day 28, no deaths (0/6) occurred in the Trunc‐LMP2A‐RNP group (Figure S7C, Supporting Information).

### 2.5 Trunc‐LMP2A‐RNP Induces A More Potent Immune Response Than FL‐LMP2A‐RNP in Mice with Tumors

**Figure 4:**
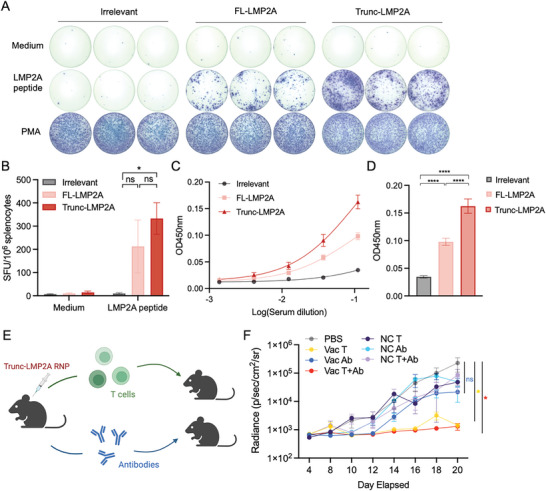
Activation of humoral and cellular immune responses by Trunc‐LMP2A‐RNP and LMP2A‐FL‐RNP in tumor‐bearing mice. A,B) T‐cell responses against LMP2A were determined using the IFN‐γ ELISPOT assay. T cells were isolated from tumor‐bearing mice on day 28 in Figure 3 and stimulated with LMP2A peptides. A) Representative figures and B) frequencies are illustrated. Sample size: Irrelevant (*n =* 6); FL‐LMP2A (*n =* 8); Trunc‐LMP2A (*n =* 12). (The sample sizes of the irrelevant and FL‐LMP2A groups were reduced due to mice mortality). C,D) Measurement of total serum anti‐LMP2A antibodies from tumor‐bearing mice immunized with 40 µg Trunc‐LMP2A‐RNP (*n =* 12), FL‐LMP2A‐RNP (*n =* 12), or irrelevant RNP (*n =* 14) on day 20. ELISA was performed by coating 96‐well plates with LMP2A peptides, and the absorbance (optical density, OD) was evaluated at 450 nm. E) Adoptive transfer of vaccine‐elicited T cells and/or antibodies to unimmunized tumor‐bearing mice: Healthy C57BL/6 mice were vaccinated with 40 µg Trunc‐LMP2A RNP or PBS on days 1, 3, and 7, administered three times (*n =* 30). On day 13, recipient C57BL/6 mice were intravenously injected with 2 × 10^5^ B16‐LMP2A cells (*n =* 6). On day 14, T cells and antibodies were isolated from the spleen and peripheral blood of vaccinated (Vac) or unvaccinated (NC) mice, respectively, and then transferred into tumor‐bearing mice. T cells and antibodies from unvaccinated mice served as the control (NC) group (*n =* 6). (T cells: 1 × 10^7^ per mouse; antibodies: 200 µg per mouse). F) In vivo bioluminescence imaging of tumor growth in recipient mice. VAC T: T cells from vaccinated mice, VAC Ab: antibodies from vaccinated mice, VAC T + Ab: both T cells and antibodies from vaccinated mice were injected into recipient mice. NC T: T cells from control mice, NC Ab: antibodies from control mice, NC T + Ab: both T cells and antibodies from vaccinated mice were injected into recipient mice (*n =* 6). Significance was determined using one‐way ANOVA followed by Tukey's multiple comparisons (B and D) and two‐way ANOVA followed by Dunnett's multiple comparisons test (F). Error bars, mean ± SEM. *p < 0.05; **p < 0.01; ***p < 0.001; ****p < 0.0001.

To gain further insights into the underlying immune response of the antitumor effects of Trunc‐LMP2A‐RNP and FL‐LMP2A‐RNP in tumor‐bearing mice, we harvested splenocytes and measured the number of LMP2A‐specific IFN‐γ secreting splenocytes. As expected, the Trunc‐LMP2A‐RNP group produced the most intense IFN‐γ spots, while the FL‐LMP2A‐RNP group secreted moderate IFN‐γ when restimulated with LMP2A peptides (**Figure** **4**A,B). In addition, we found that mice vaccinated with Trunc‐LMP2A‐RNP and FL‐LMP2A‐RNP had increased T cell production of tumor necrosis factor (TNF)‐α and IFN‐γ (Figure S9A, Supporting Information). The enhanced secretion of TNF‐α and IFN‐γ by activated T cells indicates that Th1 responses were stimulated.^[^ ^53^, ^54^ ^]^ However, we did not observe an increase in the production of IL‐2, the main cytokine produced during the primary reaction of Th1 cells, in vaccinated mice (Figure S9A, Supporting Information)^[^ ^55^ ^]^ Furthermore, we evaluated the infiltration of T cells into the tumor microenvironment using IHC staining of CD4 and CD8. We found increased CD4+ and CD8+ T cell accumulation at the tumor sites of mice treated with Trunc‐LMP2A‐RNP, followed by those treated with FL‐LMP2A‐RNP (Figure S10, Supporting Information).

The control group demonstrated limited T‐cell infiltration, which may account for their rapid tumor progression and poor prognosis (Figure S10, Supporting Information). Furthermore, the levels of anti‐LMP2A antibodies were evaluated using ELISA. Surprisingly, Trunc‐LMP2A‐RNP vaccinated mice exhibited significantly higher LMP2A‐specific IgG antibody titers compared to those vaccinated with FL‐LMP2A‐RNP or the irrelevant control (Figure 4C,D). In addition to cellular immune responses, humoral immune responses may play a role in the anti‐tumor efficacy of the vaccines.^[^ ^56^, ^57^ ^]^ To investigate the significance of B and T cell responses in anti‐tumor activity, we conducted an adoptive transfer of vaccine‐elicited T cells and/or antibodies to assess their effectiveness against antigen‐expressing tumor cells in mice (Figure 4E). T cells and antibodies were isolated from Trunc‐LMP2A‐RNP immunized mice and transferred into B16‐LMP2A tumor‐bearing mice. We found that tumor progression was significantly inhibited in two groups: the one that received T cells from vaccinated mice (VAC T) and the one that received both T cells and antibodies from vaccinated mice (VAC T+Ab) (Figure 4F). However, we observed no significant difference between the PBS group and the group that received antibodies only (VAC Ab), as well as the other control groups (NC T, NC Ab, and NC T+Ab) (Figure 4F).

Therefore, the enhanced LMP2A‐specific T cell and B cell responses observed in Trunc‐LMP2A‐RNP vaccinated mice suggests that this vaccine may be more effective than FL‐LMP2A‐RNP in inducing an immune response against LMP2A in tumor‐bearing mice, and T cell responses were mainly responsible for the anti‐tumor effectiveness.

### 2.6 Evaluation of the Anti‐Tumor Efficacy of Trunc‐EBNA1‐RNP and Trunc‐EBNA3A‐RNP in Mice

**Figure 5:**
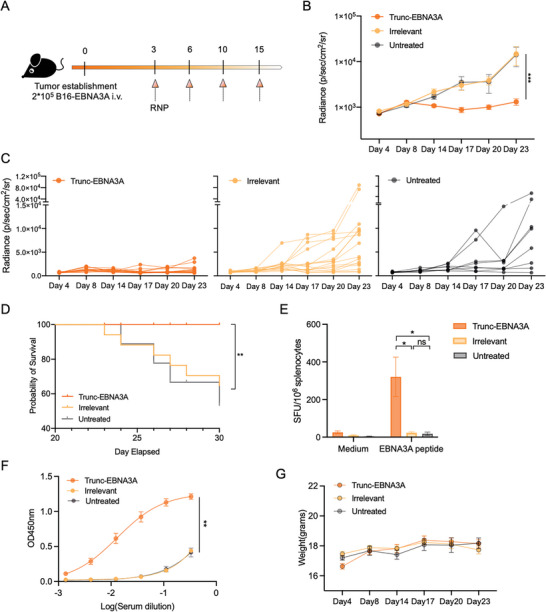
Inhibition of B16‐EBNA3A tumor growth by Trunc‐EBNA3A‐RNP in mice. A) B16‐EBNA3A cells (2 × 10^5^ per mouse) were injected intravenously into C57BL/6 mice, which were divided randomly into three groups and intravenously treated with Trunc‐EBNA3A‐RNP (*n =* 16), irrelevant‐RNP (*n =* 16), or left untreated (*n =* 8). B,C) In vivo bioluminescence imaging of B16‐EBNA3A tumor growth. B) Average bioluminescent signals of mice from three groups, and C) individual tumor growth curves (C) (left: Trunc‐EBNA3A‐RNP; middle: irrelevant‐RNP; right: untreated). D) Kaplan–Meier survival curve of tumor‐bearing mice in different groups. E) Splenocytes harvested from Trunc‐EBNA3A‐RNP, irrelevant‐RNP vaccinated, or untreated mice were stimulated with 10 µg mL^−1^ EBNA3A peptides overnight and analyzed using ELISPOT (*n =* 4). Spots were detected with an anti‐IFN‐γ antibody (1 µg mL^−1^ R4‐6A2, Mabtech). F) Enzyme‐linked immunosorbent assay (ELISA) detection of anti‐EBNA3A antibody in serum harvested from tumor‐bearing mice treated with Trunc‐EBNA3A‐RNP (*n =* 16), irrelevant‐RNP (*n =* 16), or left untreated (*n =* 8) on day 20. ELISA plates (96 wells) were coated with EBNA3A peptides overnight and blocked with 5% bovine serum albumin. G) Weight change in tumor‐bearing mice was monitored, and no significant difference existed between groups. Significance was determined using two‐way ANOVA followed by Dunnett's multiple comparisons test (B), log‐rank test (C), and one‐way ANOVA followed by Tukey's multiple comparisons (E and F). Error bars, mean ± SEM. *p < 0.05; **p < 0.01; ***p < 0.001; ****p < 0.0001.

We further evaluated the anti‐tumor efficacy of Trunc‐EBNA1‐RNP and Trunc‐EBNA3A‐RNP in a mouse tumor model using B16 cell lines stably expressing EBNA1 or EBNA3A (B16‐EBNA1 and B16‐EBNA3A) (Figure S4A, Supporting Information). For Trunc‐EBNA3A‐RNP, C57BL/6 mice were intravenously inoculated with B16‐EBNA3A cells and subsequently vaccinated with Trunc‐EBNA3A‐RNP or irrelevant RNP (**Figure** **5**A). To monitor tumor growth without any intervention, eight mice were left untreated. Tumor progression was significantly inhibited in the Trunc‐EBNA3A‐RNP group compared with those in the irrelevant‐RNP and the untreated control groups (Figure 5B,C). Additionally, tumor‐bearing mice showed improved survival using Trunc‐EBNA3A‐RNP compared to those in the irrelevant‐RNP and untreated controls (Figure 5D). No significant difference in survival or tumor burden was observed in the irrelevant RNP and untreated groups, suggesting that the irrelevant RNP did not influence tumor progression (Figure 5B–D).

Splenocytes were isolated from vaccinated mice on day 30, incubated with EBNA3A peptides, and assessed using the IFN‐γ ELISPOT assay to evaluate cellular immune response. The Trunc‐EBNA3A‐RNP group demonstrated higher IFN‐γ production than those in the irrelevant‐RNP and untreated groups, which mounted undetectable responses (Figure 5E). In addition, elevated EBNA3A‐specific antibody titers were detected in the sera from mice treated with Trunc‐EBNA3A‐RNP (Figure 5F).

**Figure 6:**
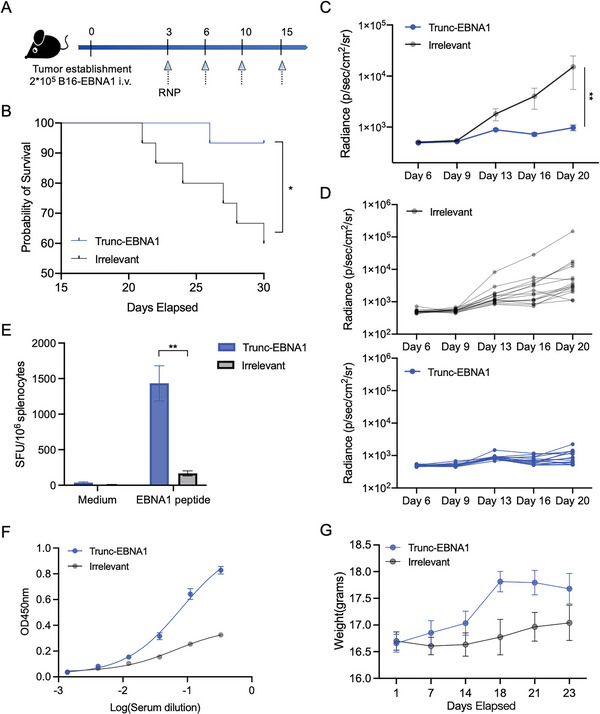
Inhibition of B16‐EBNA1 tumor growth by Trunc‐EBNA1‐RNP in mice. A) Schematic of the immune study in tumor‐bearing mice. C57BL/6 mice (6–8‐week‐old) were inoculated with B16‐EBNA1 cells (2 × 10^5^ per mouse) intravenously on day 0 and immunized i.v. with Trunc‐EBNA1‐RNP or irrelevant RNP on days 3, 6, 10, and 15 (*n =* 15). B) Kaplan–Meier survival curve of B16‐EBNA1‐bearing mice treated with Trunc‐EBNA1‐RNP or irrelevant RNP. C,D) Bioluminescence imaging was performed to monitor B16‐EBNA1 tumor growth in vivo. C) The average bioluminescent signals of mice from the two groups are shown in, and D) the tumor growth curves of individual mice are shown in (upper: irrelevant RNP; lower: Trunc‐EBNA1‐RNP). E) ELISPOT was used to detect IFN‐γ‐secreting splenocytes specific to EBNA1 peptides. Splenocytes harvested from vaccinated mice were stimulated with 10 µg mL^−1^ EBNA1 peptides overnight, and the medium alone served as the negative control (*n =* 5). F) Detection of EBNA1‐specific antibody in mice sera (*n =* 15). Levels of anti‐EBNA1 antibodies were measured using ELISA. G) Body weight of tumor‐bearing mice was monitored (*n =* 15). Although the body mass increased marginally in the vaccinated mice, no significant difference was found between the two groups. Significance was determined using the log‐rank test (B), two‐way ANOVA and Dunnett's multiple comparisons tests (C and F), and Mann–Whitney U test (E). Error bars, mean ± SEM. *p < 0.05; **p < 0.01; ***p < 0.001; ****p < 0.0001.

We evaluated the protective effect of Trunc‐EBNA1‐RNP in a mouse tumor model by injecting 2 × 10^5^ B16‐EBNA1 cells intravenously into C57BL/6 mice (**Figure** **6**A). The administration of Trunc‐EBNA1‐RNP significantly suppressed tumor development and improved the survival time of B16‐EBNA1‐bearing mice (Figure 6B–D). Furthermore, potent EBNA1‐specific cellular and humoral immune responses were elicited by Trunc‐EBNA1‐RNP in mice (Figure 6E,F). However, the irrelevant‐RNP group also showed a weak antibody response, likely due to EBNA1 expression by B16‐EBNA1 cells (Figure 6F).

To further investigate the cytokine responses elicited by Trunc‐EBNA1‐RNP and Trunc‐EBNA3A‐RNP, splenocytes were collected and analyzed using flow cytometry upon EBNA1 or EBNA3A peptides stimulation. The results showed that proportions of interleukin (IL)−2, TNF‐α‐, and IFN‐γ‐positive T cells increased in mice treated with either Trunc‐EBNA1‐RNP or Trunc‐EBNA3A‐RNP, indicating that both vaccines triggered Th1 immune responses (Figure S11A,B, Supporting Information).^[^ ^53^, ^54^ ^]^ Furthermore, Trunc‐EBNA3A‐RNP increased IL‐4 production in both CD4+ and CD8+ T cells. However, the elevation of IL‐4‐producing cells was not statistically significant in mice treated with Trunc‐EBNA1‐RNP, possibly due to the relatively small sample size and heterogeneity in the mice (Figure S11A,B, Supporting Information). Thus, both Trunc‐EBNA1‐RNP and Trunc‐EBNA3A‐RNP can elicit Th1 immune responses, which contribute to their anti‐tumor efficacy.^[^ ^58^, ^59^ ^]^

## 3 Discussion

In this study, we developed and tested three mRNA vaccines expressing the T‐cell‐epitope‐rich domain of EBV latent proteins, including Trunc‐LMP2A, Trunc‐EBNA1, and Trunc‐EBNA3A. Our results demonstrated that these vaccines could effectively activate both T‐cell and B‐cell immune responses in mice, thus inhibiting tumor progression and improving survival in tumor‐bearing mice. Our findings suggest that mRNA‐based vaccines targeting the T‐cell‐epitope‐rich domain of EBV latent proteins could be an attractive therapeutic strategy for treating EBV‐associated malignancies.

EBV is a common herpesvirus associated with various types of cancers, causing significant morbidity and mortality worldwide.^[^ ^23^ ^]^ Despite the development of various therapeutic EBV vaccines, none of these have been clinically approved.^[^ ^24^, ^60^, ^61^ ^]^ mRNA‐based vaccines have emerged as a promising new approach in preventing severe acute respiratory syndrome coronavirus 2 and have recently been tested for tumor immunotherapy in prostate cancer, melanoma, and non‐small cell lung cancer.^[^ ^30^, ^62^, ^63^ ^]^ Moderna started the phase I clinical trial of EBV vaccine mRNA‐1189, encoding EBV envelope glycoproteins (gH, gL, gp42, gp220), aiming at preventing EBV infection (NCT05164094). In this study, we developed the following mRNA‐based therapeutic EBV vaccines: Trunc‐LMP2A‐RNP, Trunc‐EBNA1‐RNP, and Trunc‐EBNA3A‐RNP and evaluated their immunogenicity and effectiveness in vivo.

EBV latent antigens contribute to the tumorigenic potential of EBV through various mechanisms.^[^ ^16^, ^19^, ^64^, ^65^ ^]^ To improve safety, the functional residues of LMP2A and the NLSs of EBNA1 and EBNA3A were either avoided or mutated. Meanwhile, most immunodominant T‐cell epitopes identified using T‐cell assays were preserved.^[^ ^38^, ^39^ ^]^

Our results demonstrated that Trunc‐LMP2A‐RNP, Trunc‐EBNA1‐RNP, and Trunc‐EBNA3A‐RNP induced strong antigen‐specific cellular responses in healthy mice. Surprisingly, EBV‐specific immune responses were considerably enhanced using the truncated vaccines, compared to those with vaccines encoding the full‐length antigens. The rationale for this is still unclear. However, several possibilities exist. Firstly, the full‐length antigens contain regions encoding a few T cell epitopes, which may reduce the density of T cell epitopes in translated proteins and the probability of major histocompatibility complex (MHC) presentation.^[^ ^66^, ^67^, ^68^ ^]^ Secondly, truncated antigens are more efficiently expressed than their full‐length counterparts, which results in the presentation of a greater number of epitopes by the antigen‐presenting cells (APCs).^[^ ^69^ ^]^ Thirdly, the cytoplasmically expressed NLS‐mutated EBNA1 and EBNA3A might more efficiently interact with the transporter associated with antigen processing (TAP) and presentation on MHC molecules than that by nuclear‐expressed antigens.^[^ ^70^ ^]^ However, the exact mechanism requires further investigation.

Also, we observed that specific cellular immunity was not induced in mice vaccinated with FL‐EBNA1‐RNP. This finding is consistent with those of previous reports that the Gly‐Arg‐rich region of EBNA1 inhibits antigen processing and MHC presentation.^[^ ^71^, ^72^ ^]^ In addition, our study suggests that administering four doses of the vaccine offers superior protection than that with three doses (data not shown) and speculates that the protective effect could be further enhanced with additional doses of the vaccine. Notably, 3–7 doses were utilized in the previous studies.^[^ ^51^ ^]^

Furthermore, we observed that the Trunc‐LMP2A‐RNP showed a more potent antitumor effect in the tumor‐bearing mouse model than that by FL‐LMP2A‐RNP. While the average tumor bioluminescence signal in the two groups of mice showed no significant difference, the Trunc‐LMP2A‐RNP vaccinated mice exhibited enhanced survival. This phenomenon may be due to several reasons. Firstly, there was less tumor burden in the Trunc‐LMP2A‐RNP treated group, but the sample size was insufficient to detect a significant difference. Secondly, rebound tumor growth in mice treated with LMP2A‐FL‐RNP was faster and led to death in a relatively short time, which may have masked the difference in tumor growth in the late stages. Finally, the host immune status may also be associated with survival, and Trunc‐LMP2A‐RNP could elicit more robust anti‐tumor immune responses, which may be associated with improved general health and survival rates in mice.^[^ ^73^, ^74^ ^]^

Therapeutic EBV vaccines have been studied extensively, with some showing potential effectiveness.^[^ ^24^, ^25^, ^61^, ^75^, ^76^ ^]^ For example, in phase I clinical trials, autologous DCs pulsed with LMP‐2 peptides elicit EBV‐specific immune responses in patients with NPC.^[^ ^24^, ^61^ ^]^ Similarly, a study in the UK tested modified vaccinia Ankara (MVA) expressing EBNA1 and LMP2A fusion protein (MVA‐EL) in EBV‐positive patients(patients with cancer) and generated EBV‐specific T‐cell responses in treated patients.^[^ ^25^ ^]^ Another study showed that mature DCs loaded with FLRGRAYGL, an EBNA3A peptide, elicit anti‐EBV‐specific CTLs against antigen‐pulsed B lymphoblastoid cell lines (BLCLs)^[^ ^76^ ^]^ Additionally, adenovirus vaccines, such as recombinant serotype 5 adenoviruses (rAd5) encoding LMP‐2 (rAd5‐EBV‐LMP2), have been used in phase I clinical trials and have shown an increase in circulating CD3+CD4+ cells.^[^ ^75^ ^]^

However, despite significant progress in developing therapeutic EBV vaccines, the survival rates have not improved considerably in patients with NPC or other EBV‐related malignancies using these approaches, and there are currently no approved vaccines.^[^ ^24^ ^]^ This may be due to the complex immune escape ability of EBV, insufficient patients included in clinical trials, or the inefficacy of previous vaccine methods. For example, the clinical efficacies of most DC‐based therapies remain suboptimal, which may be due to the widespread intratumor immunosuppressive profile and technical limitations in using monocyte‐derived DCs. Moreover, DC‐based therapy is limited by the ability to deliver a limited number of epitopes, high cost, and labor requirements for production.^[^ ^23^, ^27^ ^]^

In contrast, recombinant viral vector vaccines can potentially provide a wide range of epitopes, but the immune response to the vector itself can be a limitation for repeated administration or patients with pre‐existing immunity.^[^ ^77^, ^78^, ^79^, ^80^ ^]^

In this study, mRNA‐based EBV therapeutic vaccines were developed, and their efficacy and safety were demonstrated in mouse tumor models. Future studies are necessary to assess the safety, immunogenicity, and anti‐tumor effectiveness of the vaccines in other models, including humanized mouse models. While mouse models are commonly used to study immunological responses and evaluate the efficacy and safety of potential therapies, they are not perfect models for human disease. One limitation is the variation between human and mouse immune systems. As a result, the immune response observed in a mouse model may not fully reflect the response in humans. Additionally, mouse models often lack the complexity of the human immune system and may not fully recapitulate the microenvironment of human tumors.^[^ ^81^, ^82^ ^]^ Despite these limitations, mouse models remain an important tool for the preclinical evaluation of potential therapies, including vaccines. In this study, the immunogenicity of antigen truncations was predicted using human T‐cell epitopes. We also compared the human and mouse epitopes in the Immune Epitope Database (IEDB) and found there were only four known epitopes for EBNA1 and LMP2A. Among these, “TYGPVFMCL” and “VYGGSKTSL” were shared with human epitopes. All four epitopes were included in the Trunc‐EBNA1 or Trunc‐LMP2A sequences. While human leukocyte antigen (HLA) transgenic mice can evaluate the MHC‐restricted immune response, cross‐species incompatibility between mouse and human antigen‐processing and presentation machinery exists, which may result in an unnatural effect on the immune response.^[^ ^83^, ^84^ ^]^ Additionally, although human CD34+ reconstituted mice can be reconstituted with human immune cells, HLA is not expressed on thymic epithelial cells, which could limit HLA‐based T‐cell education.^[^ ^85^ ^]^

Notably, the mRNA used in this study was not pseudouridine‐modified. Unmodified mRNA vaccines targeting the spleen can induce a potent T‐cell response.^[^ ^51^ ^]^ However, if the mRNA is pseudouridine‐modified and targeted to the spleen, it may reduce its immunogenicity while enhancing its expression. However, this modification could potentially lead to immune tolerance and may be useful as a vaccine for preventing autoimmune diseases.^[^ ^86^ ^]^ Despite this, there are some limitations of mRNA‐based vaccines, such as the relatively short expression time and the need for repeated doses within a short time frame. Further research should also focus on how to extend the half‐life of mRNA, prolong its expression timeframe, increase its efficiency, and reduce the frequency of administration.

Besides the three antigens reported in this study, other EBV antigens expressed in EBV‐associated cancer cells may also be promising antigens, and the combination of different EBV antigens in therapeutic vaccines for various EBV‐related diseases warrants further investigation.^[^ ^87^ ^]^ Furthermore, combining EBV therapeutic vaccines with PD‐1/PD‐L1 blocking antibodies may increase their anti‐tumor efficacy, which should be further explored.^[^ ^88^, ^89^ ^]^

In conclusion, the mRNA‐based EBV therapeutic vaccines developed in this study provide a promising new strategy for treating EBV‐related malignancies. However, further research is needed to assess their safety and efficacy in clinical trials and optimize their design to enhance their therapeutic potential. Overall, our work provides a new strategy for treating EBV‐related malignancies, and further research on EBV therapeutic vaccines is needed.

## 4 Experimental Section

### Animal Experiments

Mice were purchased from Zhejiang Vital River Laboratory Animal Technology Co., Ltd and housed in specific‐pathogen‐free (SPF) conditions at the animal research center of Sun Yat‐sen University Cancer Center. For immunization, the vaccine was administered via the tail vein in a volume of 200 µL. To establish the syngeneic allograft tumor mouse model, cells in 200 µL sterile phosphate‐buffered saline (PBS) were injected subcutaneously or in the tail vein. Mice showing poor mobility, weight loss of 20% of the baseline, or severe reduction in general health status were promptly euthanized. CO_2_ inhalation was used for euthanasia.

### Ethics Statement

All animal experiments were conducted with prior approval from the Committee on the Ethics of Animal Experiments of Sun Yat‐sen University Cancer Center (SYSUCC, approval number: L102012021020P). All experiments in this study were conducted in accordance with the ARRIVE (Animal Research: Reporting of In Vivo Experiments) guidelines.

### Plasmids

The genes encoding EBV antigens were synthesized by Genscript Biotech, Nanjing, China, and subsequently cloned into the pUC57 plasmid with a T7 promoter, 5′UTR at the N‐terminal, and 3′UTR and a 120‐poly‐A tail at the C‐terminal, to serve as mRNA templates.^[^ ^90^ ^]^ C‐terminal flag tags were added to variants of the EBV proteins for detecting expression. Flag tags were subsequently removed for the animal experiments. The ClonExpress MultiS One‐Step Cloning Kit was used to introduce point mutations. All plasmid sequences were verified using Sanger sequencing. Previously reported T‐cell epitopes shown in Table S1 (Supporting Information) were obtained from IEDB (http://iedb.org/). Transient expression plasmids for LMP2A, EBNA1, and EBNA3A fragments were constructed on the pCAGGS vector, while the pLVX vector was used for stable cell line construction.

### Generation of In Vitro Transcription (IVT) RNA

The pUC57‐based plasmids encoding the T7 promoter followed by the 5′‐UTR, open reading frame, 3′‐UTR, and a 120‐poly(A) tail were linearized using the BsmBI‐v2 restriction enzyme and subsequently purified through ethanol precipitation. The sequences of the 5′‐UTR and 3′‐UTR are listed in Table S2 (Supporting Information).^[^ ^91^, ^92^ ^]^ The HiScribe T7 RNA Kit (NEB, E2050S) was utilized to generate uncapped mRNA transcripts. The cap structure was added later with Vaccinia Capping Enzyme (NEB M2080) and mRNA Cap 2´‐O‐Methyltransferase (NEB M0366) in a single step. RNA was purified with the Monarch RNA Cleanup Kit (NEB, T2050L), followed by elution in 30 µL RNase‐free water. The purity and concentration of the purified mRNA were evaluated using agarose gel electrophoresis and NanoDrop One Spectrophotometer (Thermo Scientific). As a control, irrelevant mRNA encoding enhanced green fluorescent protein (EGFP) was used throughout immunization. The mRNA was stored for a maximum of 8 weeks at −80 °C until use.

### Liposome Composition and Preparation of the mRNA Vaccine

Liposomes composed of DOTMA (purchased from Avanti Polar Lipids, 890898P) and DOPE (obtained from Sigma‐Aldrich, 76548) were prepared as previously described.^[^ ^91^ ^]^ Briefly, lipids were dissolved in absolute ethanol and mixed at a molar ratio of 1:2 (DOPE: DOTMA). The lipid solution was added dropwise in water to form a liposome solution, which was then stirred at 200 rpm at 25°C for 1 h. The RNP complex was formulated by mixing the mRNA and liposome solutions.^[^ ^51^ ^]^ The size and zeta potential of the RNP particle were measured using a Malvern Zetasizer Nano ZS dynamic light scattering instrument (Malvern Instruments, Worcestershire, UK).

### Cell Lines

For culturing 293T cells, Dulbecco's modified Eagle medium (GIBCO Cat#C11995500BT) was used. B16 and 4T1 cells were maintained in Roswell Park Memorial Institute (RPMI) 1640 medium (GIBCO Cat# C11875500BT). The medium for all cell lines was supplemented with 10% fetal bovine serum (FBS) (GIBCO Cat#10099141). B16 cells were firstly transduced with lentivirus expressing the firefly luciferase (fLuc) and superfold green fluorescent protein (sfGFP). To minimize the immunogenicity of GFP and fLuc,^[^ ^93^, ^94^ ^]^ the expression of both transgenes is controlled by a weak but stable polyubiquitin C (UbC) promoter (lentivirus generously provided by Lin Tian at SYSUCC). Subsequently, B16 cells, which were already expressing firefly luciferase (fLuc) and superfold green fluorescent protein (sfGFP) following lentiviral transduction, were further engineered to achieve stable expression of Epstein‐Barr virus (EBV) antigens using pLVX lentivirus infection. pLVX lentivirus vectors were generated by 293T cells, which were transfected with the following plasmids: pLVX‐EBV antigen (LMP2A, EBNA1, or EBNA3A), psPAX2, and pMD2.G at a mass ratio of 2:1:1 using polyethyleneimine (PEI). Two days after transfection, the supernatant was harvested and filtered with 0.22 µm membranes, precipitated with PEG8000 NaCl solution, and resuspended in RPMI 1640. The lentivirus was kept at −80 °C until use. Cell proliferation was analyzed using the 3‐[4,5‐dimethylthiazol‐2‐yl]−2,5 diphenyl tetrazolium bromide (MTT) assay, as previously described.^[^ ^95^ ^]^ Streptomycin (100 µg mL^−1^) and penicillin (100 U mL^−1^) were added, and all the cells were cultured in a humidified incubator at 5% CO_2_ and 37 °C. Puromycin (1 µg mL^−1^) was added to select and maintain stable cell lines expressing EBV antigens.

### IFN‐γ ELISpot

IFN‐γ ELISPOT assays were performed using 96‐well plates pre‐coated with anti‐mouse IFN‐γ monoclonal antibody (mAb) (3321‐4HPW, Mabtech). The plates were washed four times with 200 µL of Dulbecco's phosphate‐buffered saline (DPBS) and then blocked with complete RPMI 1640 before use. Single‐cell suspensions were prepared from mice spleen on day 30, and 2–5×10^5^ cells were added per well. Splenocytes were incubated overnight with LMP2A, EBNA1, or EBNA3A peptide pools at final concentrations of 10 µg mL^−1^ per peptide in complete RPMI at 37 °C. Peptide pools covering EBNA1, LMP2A, or EBNA3A were synthesized by Genscript Biotech. Spots were detected according to the manufacturer's instructions using the AID ELISpot Reader.

### Immunofluorescent Staining and Flow Cytometry

Freshly isolated splenocytes (2 × 10^6^) were cultured overnight at 37 °C in 96‐well plates with LMP2A, EBNA1, or EBNA3A peptide pools (10 µg mL^−1^/peptide) in complete RPMI 1640 medium. On the following day, cells were harvested, washed with PBS, and stained with a viability dye (BD Horizon Fixable Viability Stain 700, cat. #564997) for 15 min. After washing with PBS, cells were incubated for 20 min with 100 µL stain buffer (BD Pharmingen, cat. #554656) containing 2 µL Mouse BD Fc Block (cat.#553142). Surface markers were stained at a concentration of 1:100 for 30 min on ice using the following antibodies: BB700 Rat Anti‐Mouse CD4 (BD Horizon, cat. # 566407), FITC Rat anti‐CD3ε (BioLegend, Cat#100306), APC‐Cy7 Rat Anti‐Mouse CD45 (BD Biosciences, cat. #557659), and BV510 Rat Anti‐Mouse CD8a antibody (BD Horizon, Cat. No. 563068). After surface staining, cells were fixed and permeabilized with 1× BD Fix/Perm buffer for 20 min, washed once, and resuspended in 100 µL Perm/Wash Buffer (BD Biosciences, cat. #562574). Intracellular cytokines were stained for 30 min with Brilliant Violet 421 anti‐mouse TNF‐α antibody (BioLegend Cat.# 506328), PE anti‐mouse IL‐2 antibody (BioLegend, Cat# 503803), PE/Cyanine7 anti‐mouse IFN‐γ antibody (BioLegend, Cat# 505825), and BV786 anti‐mouse IL‐4 antibody (BD Biosciences, cat. #564006) at a concentration of 1:100 in Perm/wash buffer. Finally, cells were washed and resuspended in Stain Buffer and analyzed by using a CytoFLEX LX flow cytometer (Beckman, USA) and CytExpert 2.0 software (Beckman, USA).

For the activation markers analysis, freshly isolated splenocytes were harvested, washed with PBS, and stained with a viability dye (Zombie UV Fixable Viability Kit, BioLegend, Cat# 423107) for 15 min. After washing with PBS, cells were incubated for 20 min with 100 µL stain buffer (BD Pharmingen, cat. #554656) containing 2 µL of Mouse BD Fc Block (cat.#553142). Surface markers were stained at a concentration of 1:100 for 30 min on ice using the following antibodies: Panel 1 (T cells and NK cells): APC anti‐mouse CD69 (BioLegend, Cat# 104514), PE anti‐mouse NK‐1.1(BioLegend, Cat# 108708), APC‐Cy7 Rat Anti‐Mouse CD45 (BD Biosciences, cat. #557659), FITC Rat anti‐CD3 (BD Horizon, Cat. No. 555274), BV510 Rat Anti‐Mouse CD4 antibody (BD Horizon, Cat. No. 563106), PerCP‐Cy5.5 Rat Anti‐Mouse CD8a (BD Horizon, cat. # 551162); Panel 2 (DC cells): BB515 Rat Anti‐Mouse I‐A/I‐E (BD Horizon, cat. # 565254), Alexa Fluor 700 Rat Anti‐Mouse CD86 (BD Pharmingen, cat.# 560581), Brilliant Violet 421 anti‐mouse CD11c (BioLegend, Cat# 117343), PE anti‐mouse CD40 (BioLegend, Cat# 157506). After surface staining, cells were washed and resuspended in stain buffer and analyzed using a CytoFLEX LX flow cytometer (Beckman, USA) and CytExpert 2.0 software (Beckman, USA).

### Enzyme‐Linked Immunosorbent Assay

Immunosorbent assay plates were coated with LMP2A, EBNA1, or EBNA3A peptide pools (100 ng each well in 100 µL PBS) and incubated overnight at 4 °C. The following day, the plates were blocked with 3% bovine serum albumin (BSA) diluted in 0.1% PBST (PBS with 0.1% Tween‐20) at 37 °C for 1 h and washed three times. Further, mouse serum samples were diluted serially in 3%BSA across the plate and incubated for 1 h at 37 °C. The plates were then washed with 0.1% PBST five times and incubated with goat anti‐mouse immunoglobulin G (IgG)‐horseradish peroxidase (HRP) (ab6789) (1:10 000 diluted in blocking buffer) at 37 °C for 30 min. After washing the plates five times with 0.1% PBST, 100 µL 3,3′,5,5′‐tetramethylbenzidine substrate (Tiangen Biotech Co., Ltd., Beijing, China, Cat#PA107‐02) was added to each well and kept in the dark for five minutes; the reaction was then arrested using 1 m hydrochloric acid, and the OD450 was measured using a BioTek Epoch microplate spectrophotometer.

### Western Blot

For protein sample preparation, cells were lysed in RIPA buffer (Beyotime, Jiangsu, China, P0013B), followed by incubation at 95 °C for 5 min with 5% β‐mercaptoethanol. The lysates were subjected to sodium dodecyl sulfate‐polyacrylamide gel electrophoresis and transferred onto polyvinylidene difluoride membranes. Primary antibodies, including mouse monoclonal ANTI‐FLAG M2 (Sigma‐Aldrich, F1804), and rabbit anti‐β‐Actin (13E5) (Cell Signaling Technology, 4970S), were used to probe the membranes overnight at 4 °C. The primary antibodies were removed by washing the membranes thrice with 0.1% PBST. Subsequently, the membranes were incubated with the secondary antibody conjugated with peroxidase (1:3000) for 1 h at room temperature. The following secondary antibodies were used: goat anti‐rabbit IgG HRP (Sigma‐Aldrich, 31460), goat anti‐rat IgG HRP (Absin, abs20031), donkey anti‐sheep IgG HRP (R&D Systems, HAF016), and goat anti‐mouse IgG HRP (Sigma‐Aldrich, 32430) antibodies.

### Reverse Transcription Quantitative Polymerase Chain Reaction (RT‐qPCR)

Total RNA was extracted from the collected peripheral blood samples using TRIzol Reagent (Thermo Fisher Scientific) following the manufacturer's protocol. The concentration and purity of the isolated RNA were determined using a spectrophotometer (NanoDrop, Thermo Fisher Scientific). Next, complementary DNA (cDNA) was synthesized from the isolated RNA using a reverse transcription kit (Promega GoScript Reverse Transcription System) following the manufacturer's instructions. Quantitative PCR (qPCR) was performed using the SYBR‐Green PCR kit (ChamQ Universal SYBR qPCR Master Mix, Vazyme Biotech Co., Ltd., Nanjing, China) on the Roche LightCycler 480 II. The primer sequences used for the target cytokines (IL‐6, IFN‐α, and TNF‐α) and the reference gene beta‐actin (ACTB) were as follows: IL‐6 Forward Primer: 5′‐ CACTTCACAAGTCGGAGGCT‐3′; IL‐6 Reverse Primer: 5′‐ CTGCAAGTGCATCATCGTTGT −3′; IFN‐α Forward Primer: 5′‐ CCTGTGTGATGCAGGAACC −3′; IFN‐α Reverse Primer: 5′‐ TCACCTCCCAGGCACAGA‐3′; TNF‐α Forward Primer: 5′‐ CCCTCACACTCAGATCATCTTCT‐3′; TNF‐α Reverse Primer: 5′‐ GCTACGACGTGGGCTACAG‐3′; ACTB Forward Primer: 5′‐ TGGTTACAGGAAGTCCCTCAC −3′; ACTB Reverse Primer: 5′‐ ACAGAAGCAATGCTGTCACCTT −3′. The relative expression of IL‐6, IFN‐α, and TNF‐α were determined using the 2^‐ΔΔCt method relative to the internal control ACTB.

### Adoptive Transfer of T Cells and Antibodies

To isolate T cells, mice spleens were harvested, and the lymphocytes were separated using Mouse Lymphocyte Separation Medium (Biotech, Shenzhen, China) and Mouse CD3 T Cell Isolation Kit (BioLegend, cat. # 480031) following the manufacturer's protocol. Antibodies were purified from mouse serum using rProtein G Sepharose Fast Flow resin (Cytiva Biotechnology Co., Ltd., Hangzhou, China). The serum was passed through the resin three times, followed by washing with PBS and elution with 0.2 m Glycine (pH = 3) buffer. Subsequently, the antibody solution was centrifuged, and the glycine buffer was replaced with PBS buffer using MilliporeSigma Amicon Ultra‐2 Centrifugal Filter Units (30kDa). The concentration of purified antibodies was determined using NanoDrop One Spectrophotometer (Thermo Scientific). 1 × 10^7^ T cells and/or 200 µg antibodies were given to each recipient mouse.

### Bioluminescence Imaging

Mice were injected with 200 µL D‐luciferin potassium salt (Promega, E1601) at 15 mg mL^−1^ in PBS via retro‐orbital venous sinus and anesthetized with isoflurane inhalation. Fluorescence images were then collected using the IVIS Spectrum in vivo imaging system (PerkinElmer) with an exposure time of 30–60 s, depending on the signal intensity. The average radiance (photons/s/cm^2^/sr) within the regions of interest was quantified using IVIS Living Image Software.

### Histological and Immunohistochemistry Analysis

Formalin‐fixed paraffin‐embedded tumor tissues were cut into 3‐µm‐thick tissue sections and prepared for hematoxylin and eosin or IHC staining. For IHC staining, antigen retrieval was achieved using a pressure cooker heating in 10 mM sodium citrate buffer for 10 min, followed by blocking endogenous peroxidase activity with 3% hydrogen peroxide. The slides were then incubated overnight with primary antibodies against mouse CD4 or CD8 at 4 °C, washed with 0.1% PBST, and sequentially incubated with biotinylated secondary antibody, streptavidin‐biotin complex, and 3,3′‐diaminobenzidine.

### Statistical Analyses

The experimental data underwent minimal pre‐processing, where no specific transformations, normalization techniques, or outlier evaluation methods were applied. The results were presented as mean ± standard error of the mean. The sample size (n) for each statistical analysis varied depending on assays and is provided in the figure legends. Two‐tailed unpaired Student's t‐test or Mann–Whitney U test were used for comparing two groups. To compare more than two groups, a one‐way analysis of variance (ANOVA) followed by Tukey's multiple comparisons test was used. Tumor growth was analyzed using two‐way ANOVA followed by Dunnett's multiple comparisons test. Survival curves were compared using the log‐rank (Mantel‐Cox) test. The figure legends provide detailed descriptions of the statistical methods used for each experiment. In all cases, statistical significance was considered at a *p*‐value <0.05. The levels of significance were denoted as follows: *p < 0.05, **p < 0.01, ***p < 0.001, ****p < 0.0001. Results were considered not significant (ns) when the p‐value exceeded 0.05. Data analysis and calculation of *p*‐values were performed using GraphPad Prism 8.0 software.

## Conflict of Interest

The authors declare that patent applications have been filed covering Trunc‐EBNA1‐RNP, Trunc‐EBNA3A‐RNP, and Trunc‐LMP2A‐RNP. (Patent application number: Trunc‐EBNA3A‐RNP: CN202210150445.2, M.Z., G.Z., X.K., G.B, Z.L., and G.F. are the inventors. Trunc‐EBNA1‐RNP: CN202210253318.5, M.Z., G.Z., G.B, G.L., and G.F. are the inventors. Trunc‐LMP2A‐RNP: CN202211476298.4, M.Z., G.Z. G.F., and G.L. are the inventors.) The patent applicant is Sun Yat‐sen University Cancer Center. All other authors declare no competing interests.

## Author Contributions

G.‐X.Z., G.‐L.B., and G.‐F.L. contributed equally to this work. M.Z., Q.Z., and G.F. designed the study. G.Z., G.B., G.L., X.K., and Z.L. performed the experiments. G.Z. analyzed data. G.Z., C.S., G.F., and M.Z wrote the manuscript. All authors participated in the discussion and interpretation of the results.

## Supporting information

Supporting Information

## Data Availability

The data that support the findings of this study are available from the corresponding author upon reasonable request.;
