# Supplementary material for: mRNA‐based Vaccines Targeting the T‐cell Epitope‐rich Domain of Epstein Barr Virus Latent Proteins Elicit Robust Anti‐Tumor Immunity in Mice
Source: Adv Sci (Weinh). 2023 Oct 27;10(35):2302116. doi: 10.1002/advs.202302116 (PMC10724410; doi:10.1002/advs.202302116)
Supplement: Supplementary file 1 — Supporting Information [file ADVS-10-2302116-s001.pdf]

ADVANCED SCIENCE
Open Access

Supporting Information

for *Adv. Sci.*, DOI 10.1002/advs.202302116

mRNA-based Vaccines Targeting the T-cell Epitope-rich Domain of Epstein Barr Virus Latent Proteins Elicit Robust Anti-Tumor Immunity in Mice

Ge-Xin Zhao, Guo-Long Bu, Gang-Feng Liu, Xiang-Wei Kong, Cong Sun, Zi-Qian Li, Dan-Ling Dai, Hai-Xia Sun, Yin-Feng Kang, Guo-Kai Feng, Qian Zhong* and Mu-Sheng Zeng**

Supplementary Materials

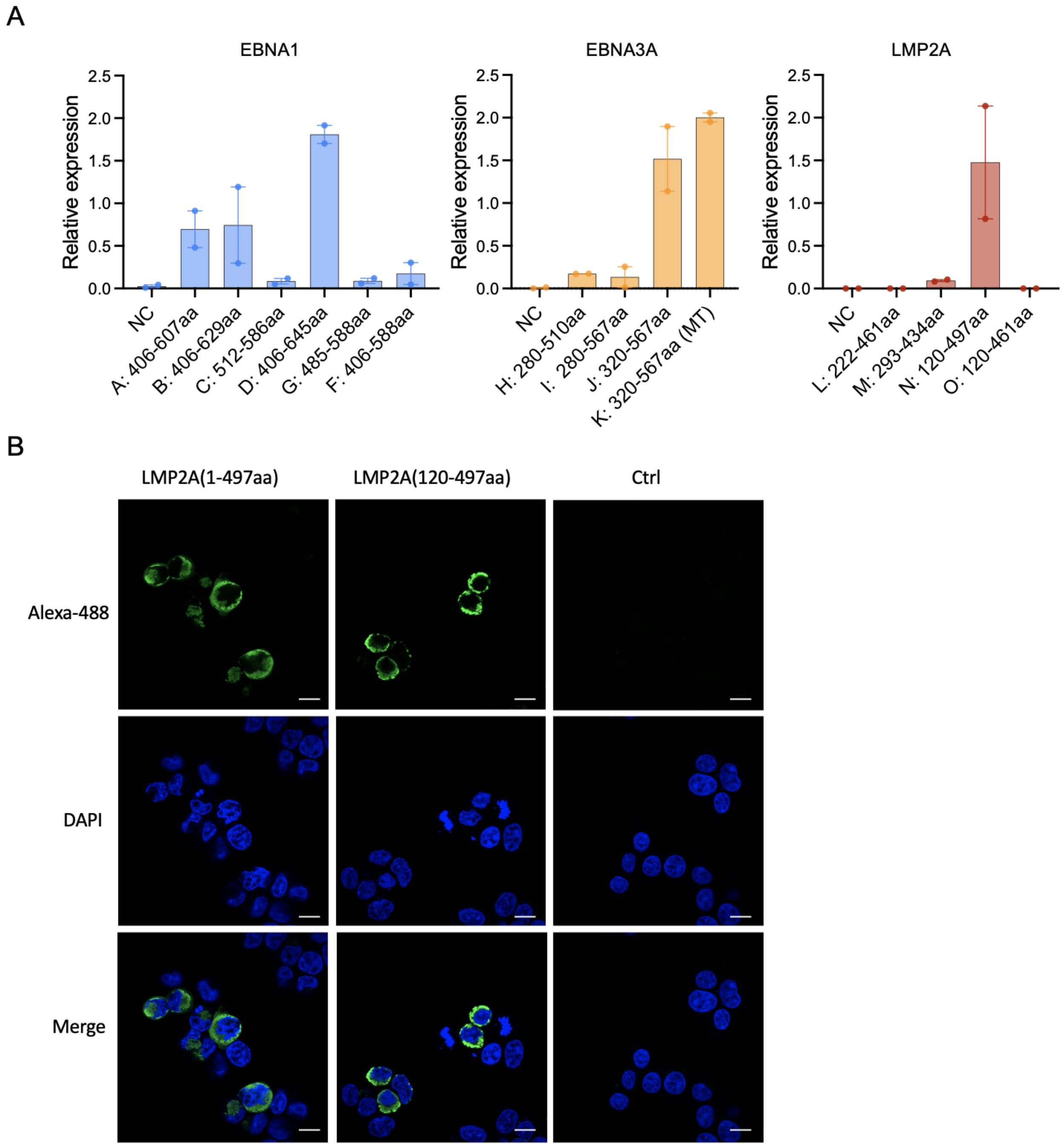

Fig.S1 Relative truncated protein expression level and expression of Flag-tagged Trunc-LMP2A and FL-LMP2A in 293T cells.

(A) Quantification of protein expression relative to beta-actin associated with Figure 1B is shown (n=2). Error bars, mean ± SEM. **(B)** Representative confocal images of 293T cells expressing flag-tagged Trunc-LMP2A(120-497aa) and FL-LMP2A(1-497aa). The corresponding mRNA was transfected into 293T cells 12 h before detection. Protein

expression was detected with Alexa Fluor® 488 conjugated anti-flag antibody(green), and the nuclei were stained with DAPI (blue). Scale Bar = 10 μm.

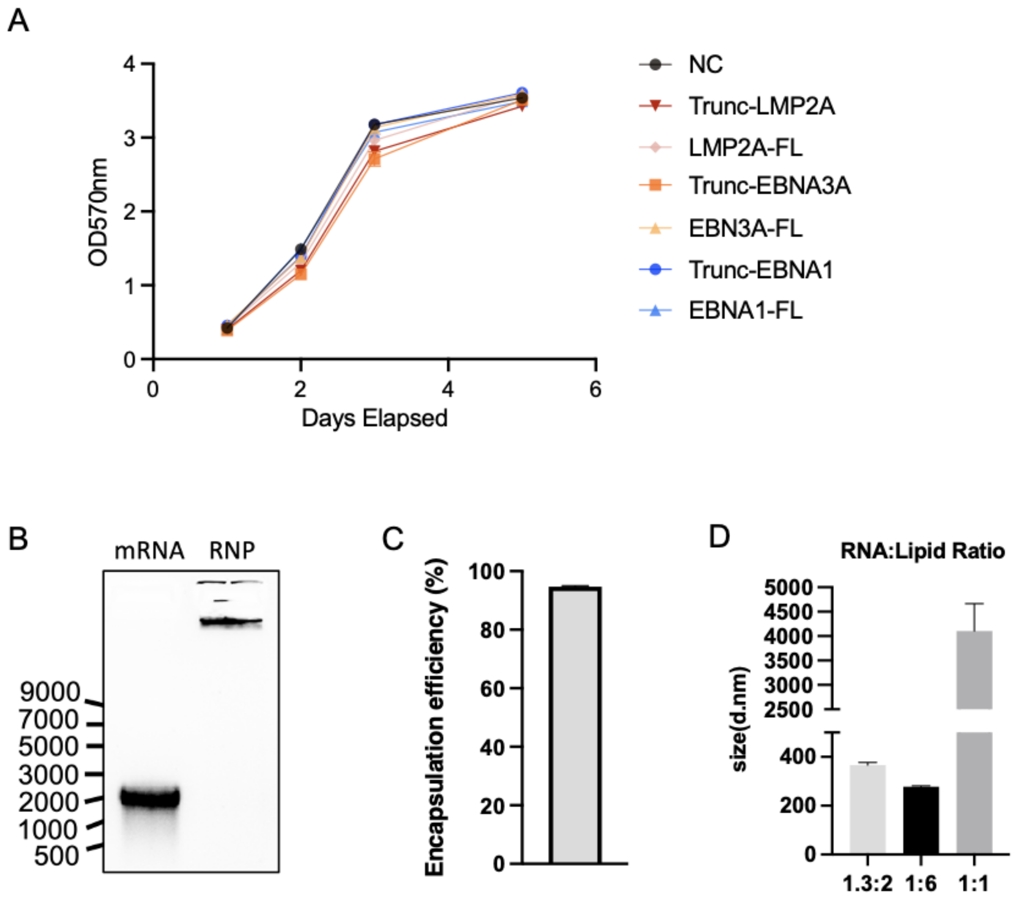

Fig.S2 Cell proliferation assay and characterization of mRNA-liposome nanoparticle complex.

(A) Cells (4T1) were seeded in 96-well plates, transfected with different antigens or an empty vector (NC) 24 h after seeding, and analyzed using the MTT cell proliferation assay. No significant difference was observed among the groups (n=6). **(B)** Trunc-LMP2A mRNA (2 μg) and mRNA complexed with liposome (Trunc-LMP2A RNP) (2 μg) were loaded on a TAE gel and ran for 20 min. (**C**) The RNP was concentrated for 60 min at 16260rcf (12,000 rpm), and the RNA concentration of the supernatant was determined. Encapsulation efficiency was calculated as the ratio of the RNA concentration in the supernatant to the total RNA concentration before liposome packaging (n=3). (**D**) The particle size of RNP was determined for different molar ratios of RNA and the cationic lipid, with a ratio of 1.3:2 being used consistently throughout the study (n=4). Error bars, mean ± SEM.

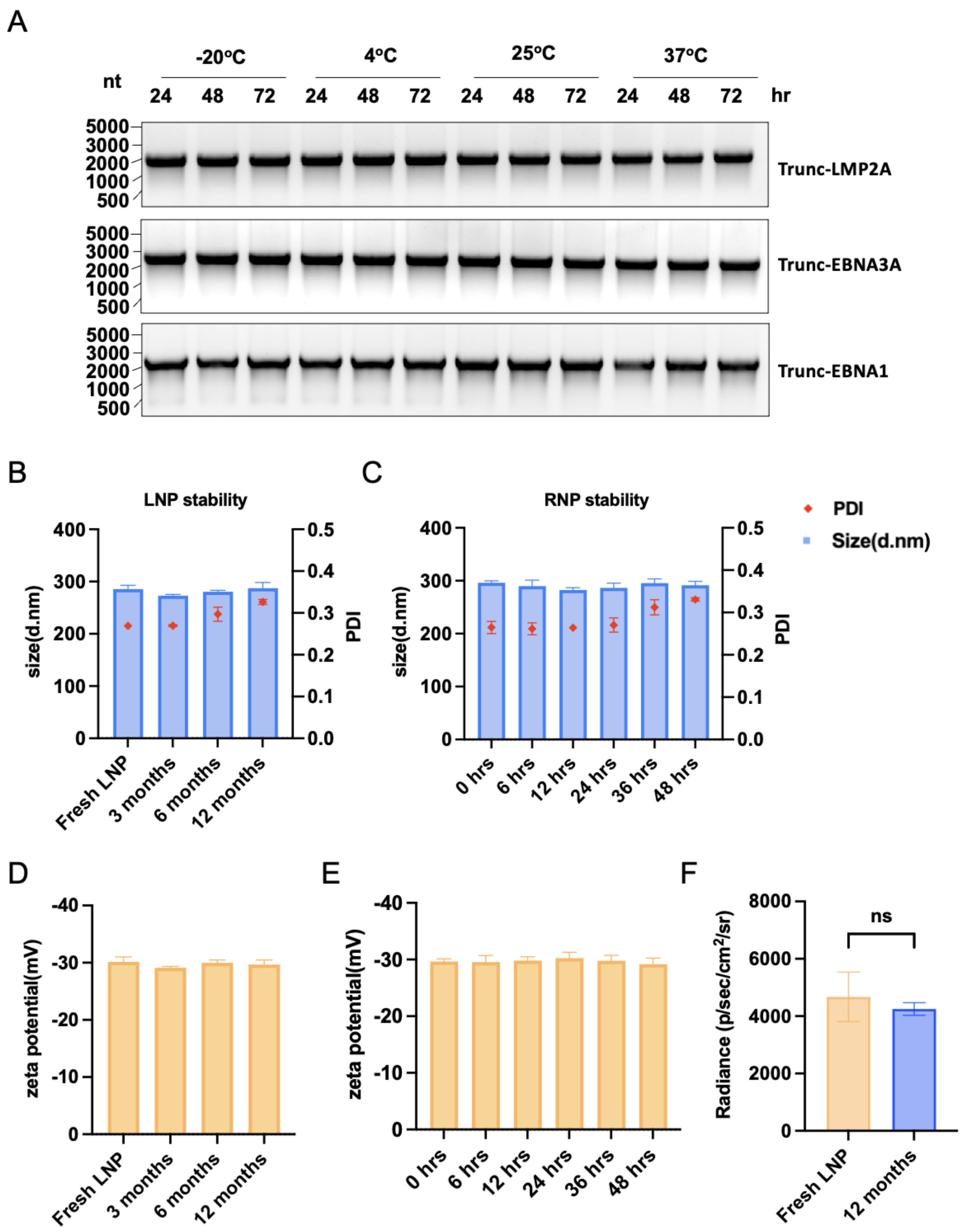

Fig. S3 Stability assessment of purified mRNA and liposomes.

(**A**) The stability of purified mRNA. Purified mRNAs encoding Trunc-LMP2A, Trunc-EBNA3A, or Trunc-EBNA1 with 5′ Cap1 and 3′ 120-poly(A) tail were stored at different temperatures for up to 3 days. mRNA was denatured in 2× RNA Loading Dye (NEB) and electrophoresed under native 1× Tris-acetate EDTA (TAE) gel conditions. (**B**) and (**D**) The particle size (**B,** blue bars), polydispersity index (PDI) (**B,** red dots) and zeta potential (**D**) of

RNP formed using freshly made liposomes or liposomes stored for 3, 6, or 12 months at 4 °C (n=3). (**C**) and (**E**) The particle size (**C,** blue bars), PDI (**C,** red dots), and zeta potential (**E**) of RNP were measured during storage from 0 to 48 h at 4 °C (n=3). (**F**) Six hours after intravenous injection of mRNA encoding luciferase complexed with either freshly made liposomes or liposomes stored for 12 months (n=3), luciferase signals were detected in C57BL/6 mice. Statistical significance was determined using a two-tailed unpaired T-test (**F**). Error bars, mean ± SEM.

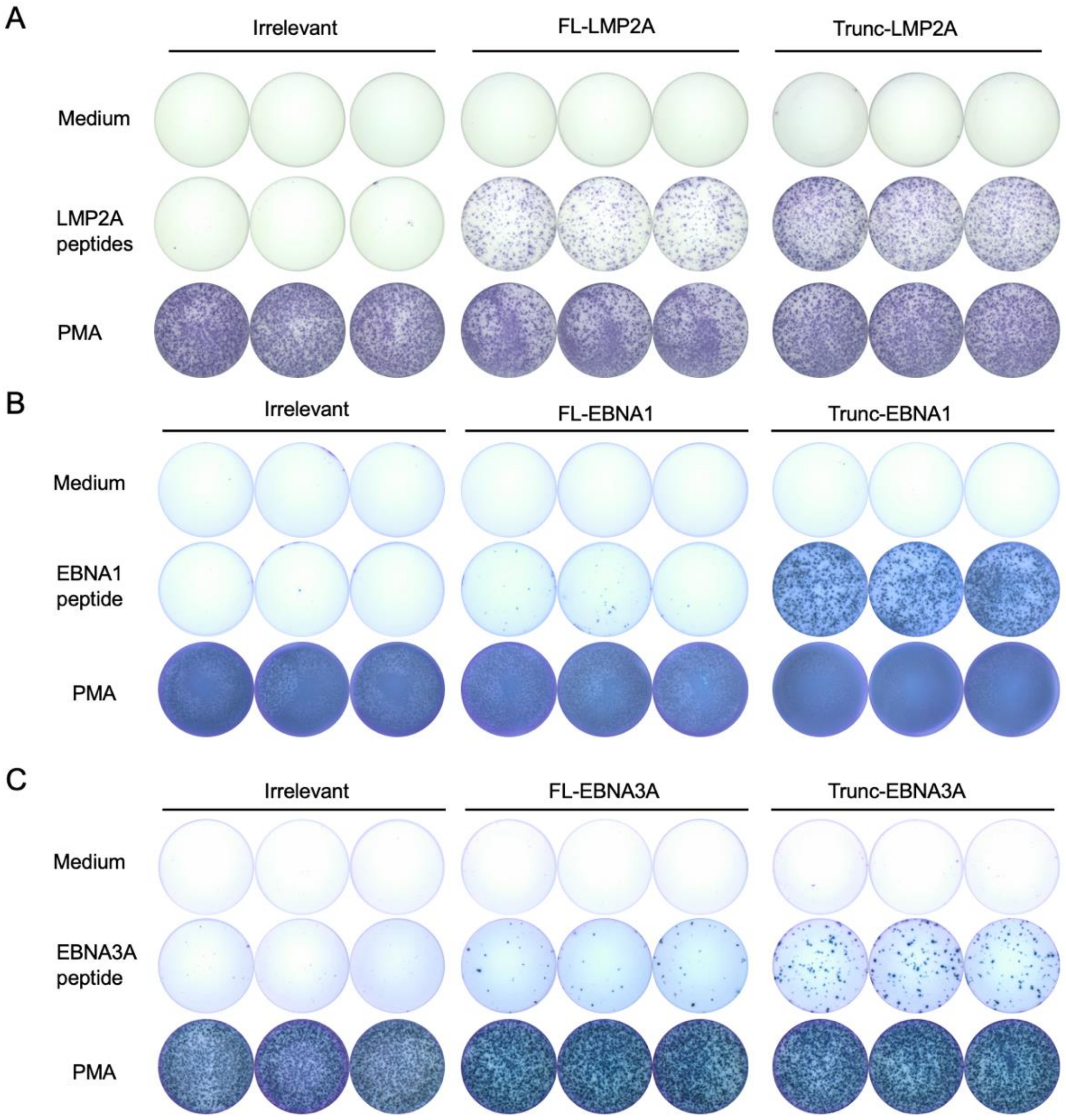

Fig. S4. RNP-elicited T cell immune responses assessed by ELISPOT.

(A-C) Representative figures of IFN-γ releasing LMP2A-specific cells **(A)**, EBNA1-specific cells (**B**), and EBNA3A-specific cells (**C**) corresponding to the ELISPOT assay are shown in Figure 2.

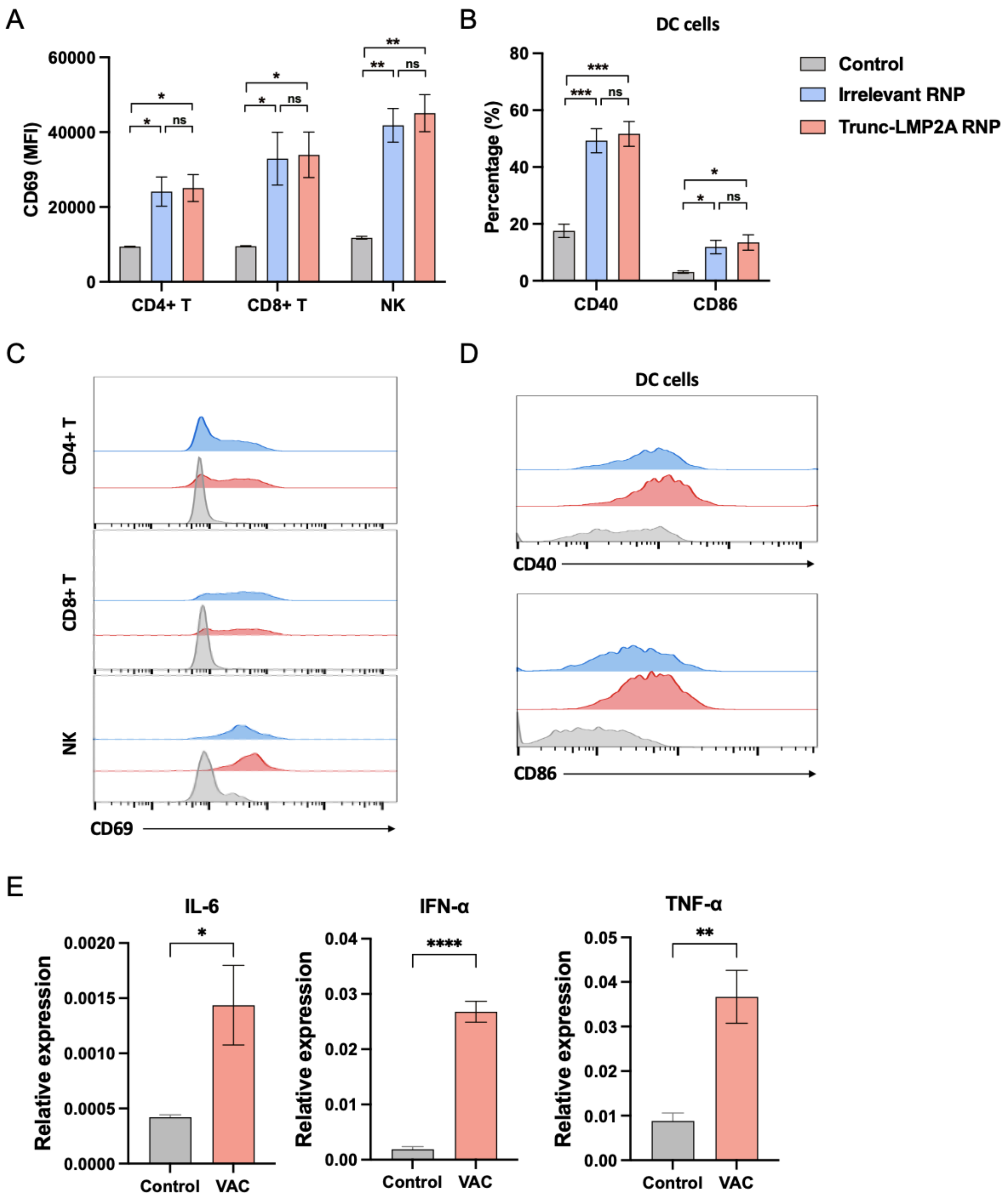

Fig.S5 Immune activation and cytokine response induced by intravenous injection of RNPs in mice.

(**A**) and (**C**) Activation markers of CD4+ T cells, CD8+ T cells, and natural killer (NK) cells were measured 24 hours after intravenous injection of 40 μg of irrelevant RNP, Trunc-LMP2A RNP, or PBS as a control in splenic immune cell subsets of C57BL/6 mice (n = 3). (**B**) and (**D**) Maturation marker of dendritic cells measured 24 h after i.v. injection of 40 μg irrelevant RNP, Trunc-LMP2A RNP, or PBS as a control in C57BL/6 mice (n=4). (**E**) Relative expression of IL-6, IFN-α, and TNF-α were measured by RT-qPCR in PBMC in the

mice 24 hours after intravenous injection of 40 µg of Trunc-LMP2A RNP or PBS as a control (n=6). Significance was determined using one-way ANOVA followed by Tukey's multiple comparisons (**A** and **B**) and two-tailed unpaired T test (**E**). Error bars, mean ± SEM. *$P < 0.05$; **$P < 0.01$; ***$P < 0.001$; ****$P < 0.0001$.

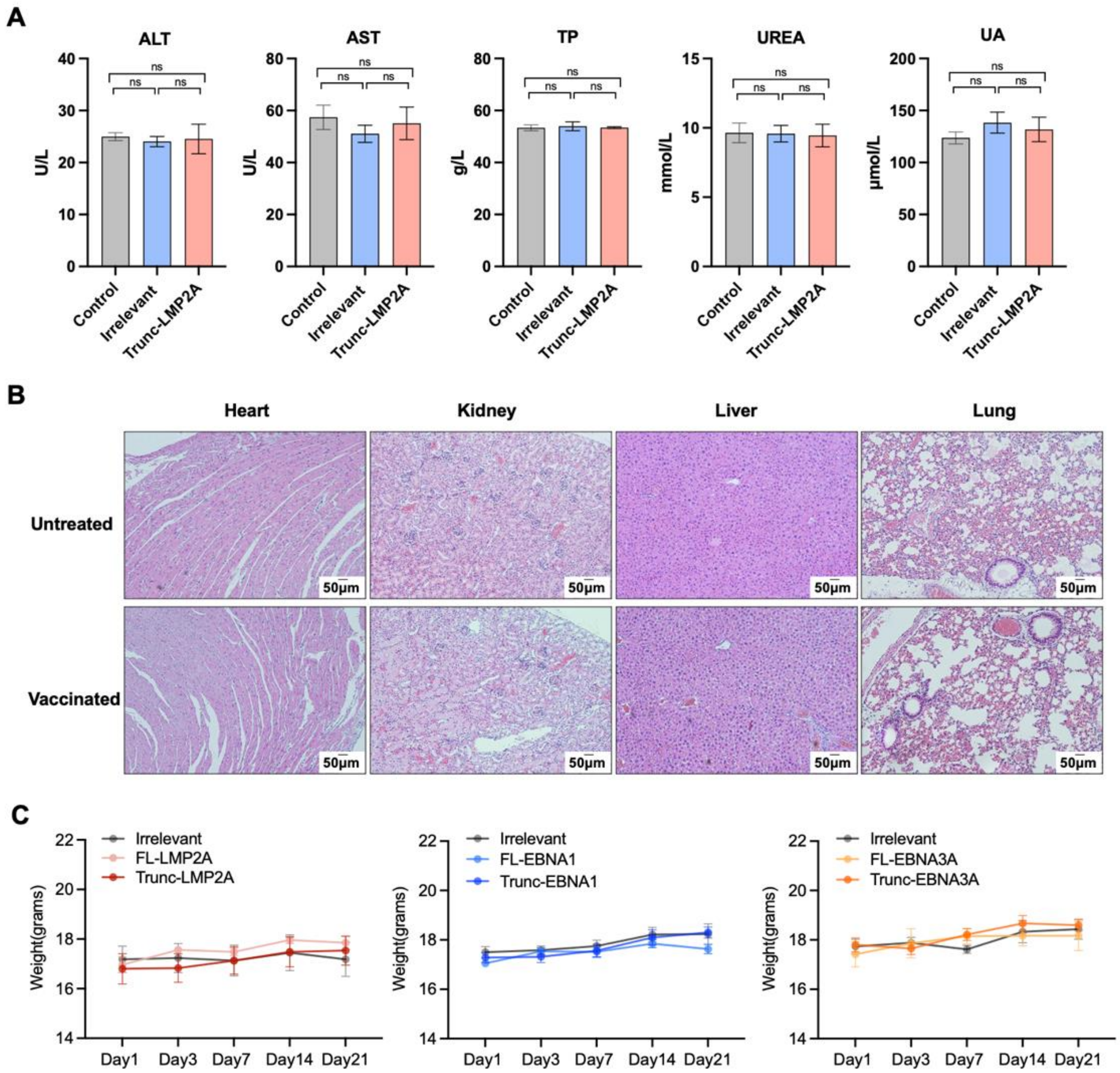

Fig.S6 No observed RNP vaccination-induced organ toxicity was assessed by biochemical assay and histological analysis.

(A) Mice were injected with 40 μg of irrelevant RNP, Trunc-LMP2A RNP, or PBS as a control, and serum alanine transaminase (ALT), aspartate aminotransferase (AST), total protein (TP), UREA, and uric acid (UA) levels were assessed on day 3 (n=3). **(B)** Representative pictures from hematoxylin eosin-stained sections of organs harvested from mice on day 21 after immunization and untreated healthy mice of the same age. No significant histological changes have occurred in vaccinated mice. Pictures presented here were from Trunc-LMP2A-RNP vaccinated and untreated healthy mice. Similarly, no significant histological changes were observed in groups treated with other RNPs (data not shown). **(C)** Body weight changes of mice treated with RNPs (n=6).

Significance was determined using one-way ANOVA followed by Tukey's multiple comparisons (**A**). Error bars, mean ± SEM.

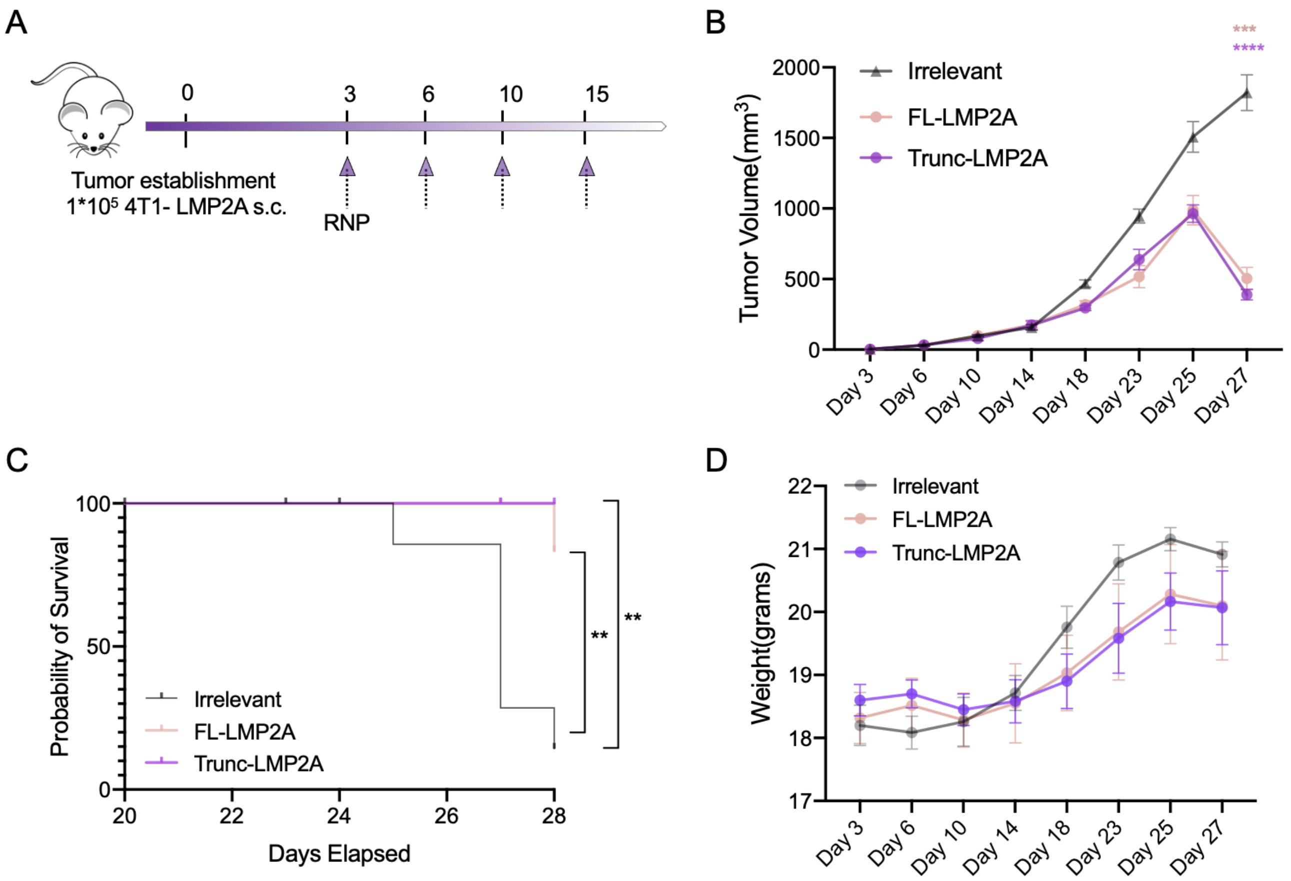

Fig.S7 Anti-tumor effects of LMP2A RNP vaccination in 4T1-LMP2A tumor-bearing mice.

(A) BALB/c mice were injected with 4T1-LMP2A cells (1 × 10^5 per mouse). Mice were divided into three groups randomly and immunized i.v. with 40 μg FL-LMP2A-RNP (n=6), Trunc-LMP2A-RNP (n=6), or irrelevant RNP (n=7) on days 3, 6, 10, and 15. **(B)** Tumor volumes were measured by blinded observers. The greatest longitudinal diameter (length) and the greatest transverse diameter (width) were determined, and tumor volume was calculated using the modified ellipsoidal formula: V = ½ (Length × $Width^2$). **(C)** Survival curves of 4T1-LMP2A-bearing mice treated with FL-LMP2A-RNP, Trunc-LMP2A-RNP, or irrelevant RNP. Mice were euthanized when the tumor volumes exceeded 2,000 mm^3 or when they reached other humane endpoints, as described in the methods. **(D)** Body weight changes of tumor-bearing mice. Significance was determined using two-way ANOVA followed by Dunnett's multiple comparisons test (**C**) and log-rank test (**D**). Error bars, mean ± SEM. *P < 0.05; **P < 0.01; ***P < 0.001; ****P < 0.0001.

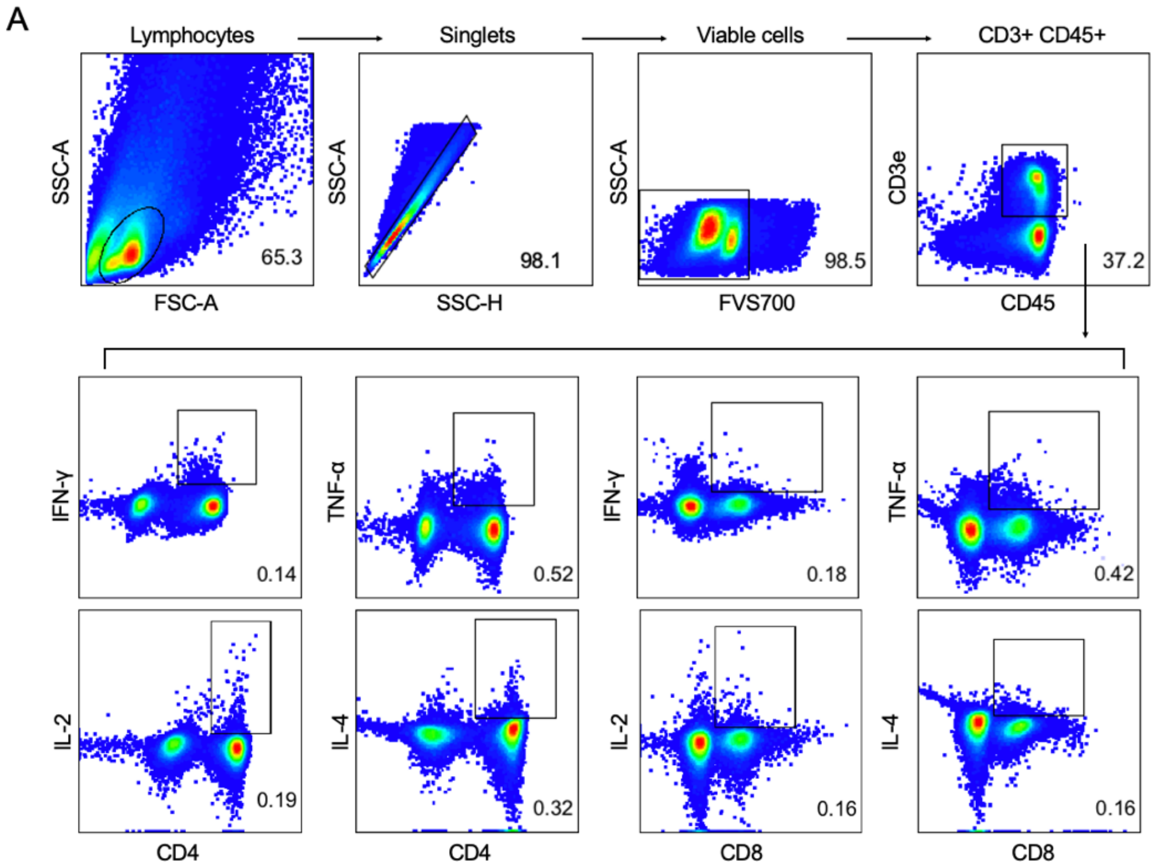

Fig.S8 Gating Strategy for Intracellular Cytokine Staining Analysis of T Cells

(A) Gating strategy for analyzing T cells in intracellular cytokine staining panel. Percentages indicate the percent of the parent population.

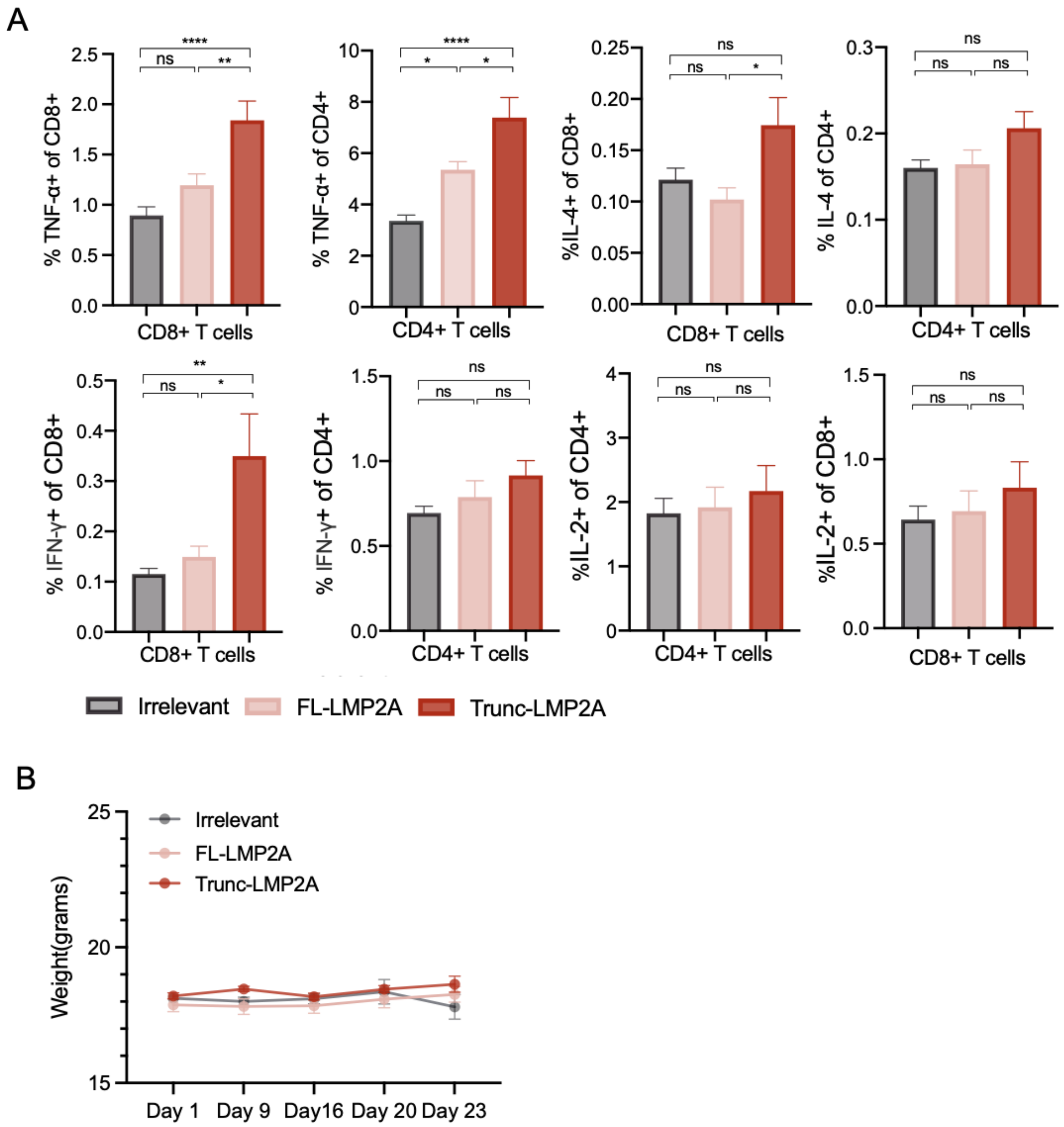

Fig.S9 Cytokine production and body weight of mice vaccinated with LMP2A RNP. (**A**)Cytokine production induced by Trunc-LMP2A-RNP, FL-LMP2A-RNP, or irrelevant RNP in mice (n=16) was assessed using flow cytometry. Splenocytes harvested from vaccinated mice were stimulated with LMP2A peptides overnight, followed by 6-h incubation with brefeldin A (BFA) (10 µg/ml). (**B**) Body weight changes of tumor-bearing mice treated with Trunc-LMP2A-RNP (n=12), FL-LMP2A-RNP (n=12), or irrelevant RNP (n=14) (related to Figure 3). Significance was determined using one-way ANOVA and Tukey's multiple comparisons (A). Error bars, mean ± SEM. *P < 0.05; **P < 0.01; ***P < 0.001; ****P < 0.0001.

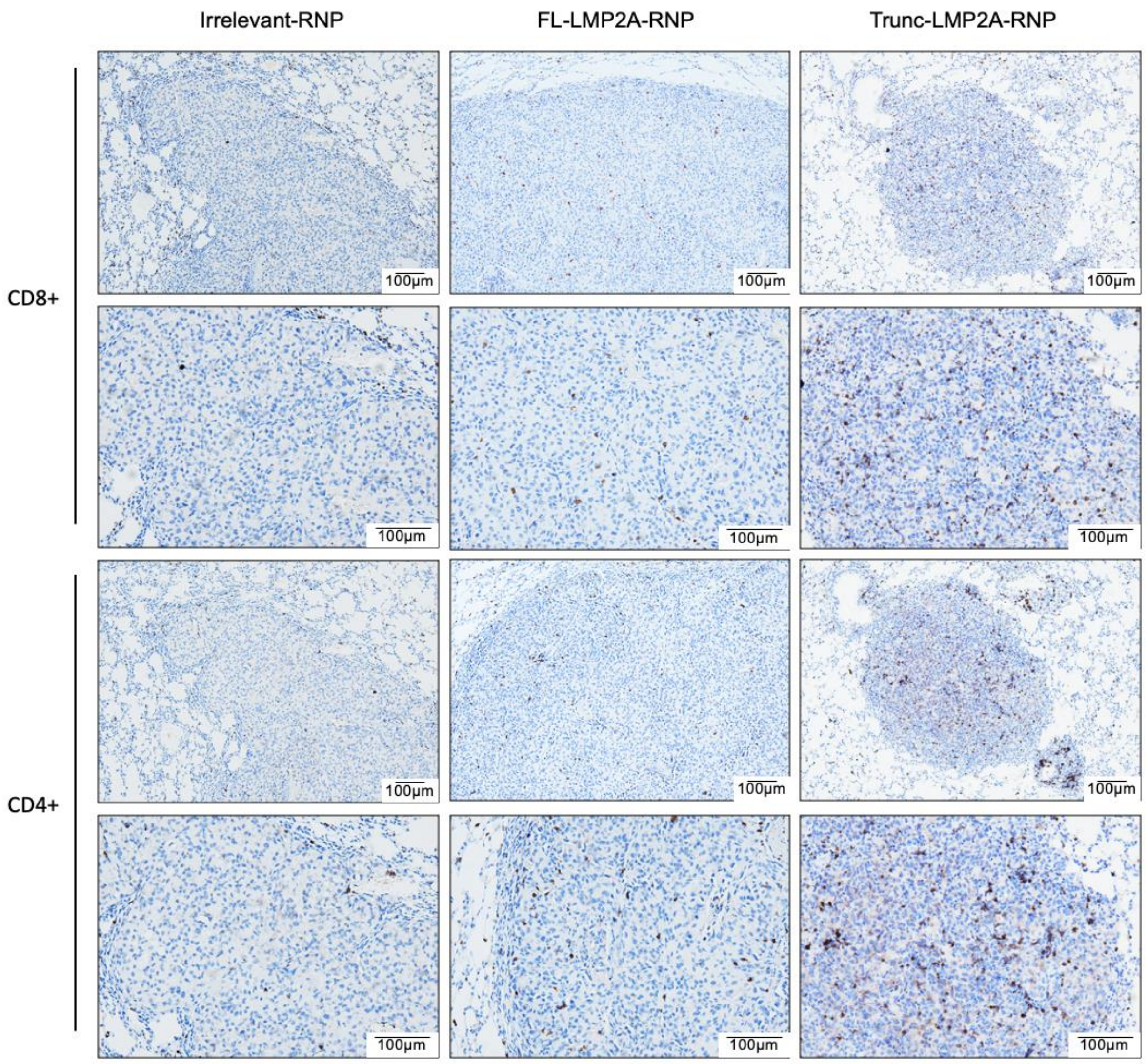

Fig.S10 Detection of CD4^{+} and CD8^{+} T-cell infiltration in tumor biopsies from mice immunized with LMP2A RNP.

Immunochemistry was used to quantify CD4^{+} and CD8^{+} T-cell infiltration in tumor biopsies from mice vaccinated with FL-LMP2A-RNP and Trunc-LMP2A-RNP, or irrelevant-RNP on day 28 post-tumor inoculation. Compared with the controls, T cell infiltration was increased in mice immunized with FL-LMP2A-RNP and Trunc-LMP2A-RNP. For most samples, Trunc-LMP2A-RNP elicits more T-cell infiltration in mice than FL-LMP2A-RNP. Representative pictures are shown. Brown regions indicate immunoreactivity. The scale bar is displayed (100 μm).

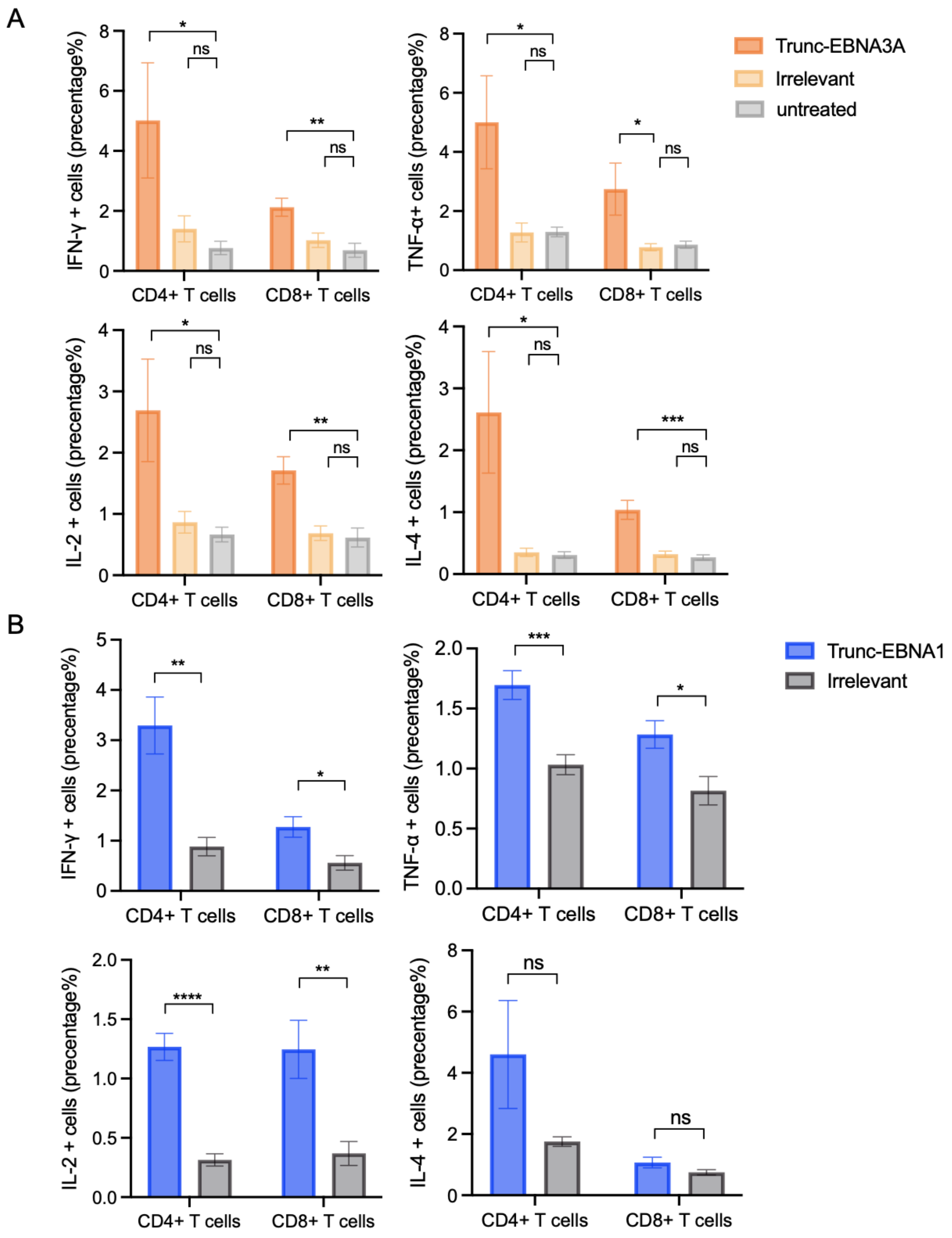

Fig.S11 Cytokine production induced by Trunc-EBNA3A-RNP and Trunc-EBNA1-RNP in tumor-bearing mice upon peptide stimulation.

(A) Splenocytes harvested from mice vaccinated with Trunc-EBNA3A-RNP, irrelevant RNP, or untreated were incubated with EBNA3A peptides overnight for flow cytometry analysis (n=6). (**B**)Splenocytes harvested from mice vaccinated with Trunc-EBNA1-RNP or irrelevant

RNP were incubated with EBNA1 peptides overnight for flow cytometry analysis (n=7). BFA (10 µg/mL was added for the final 6 h of the stimulation. The percentage of cytokine-producing cells was determined using flow cytometry. The frequency of cytokine-producing cells is shown as the mean percentage ± SEM of the parent population. Significance was determined using one-way ANOVA and Tukey's multiple comparisons (**A**) and two-tailed unpaired Student's t-test (**B**). $*P < 0.05$; $**P < 0.01$; $***P < 0.001$; $****P < 0.0001$.

Table S1 List of human T-cell epitopes in LMP2A, EBNA1, and EBNA3A (corresponding to Figure 1A)

Antigen	Abbreviation	T cell epitopes	MHC restriction	Starting Position (aa)	Number of References*	Number of Assays*
LMP2A	MGS	MGSLEMVPM	HLA-B*35:01	1	2	2
LMP2A	PYL	PYLFWLAAI	HLA-A*2402	131	10	27
LMP2A	FTA	FTASVSTVV	HLA-A*68	144	2	2
LMP2A	IED	IEDPPFNSL	HLA-B*40:01	200	13	19
LMP2A	ITV	IYVLVMLVL	HLA-A*24:02	222	4	14
LMP2A	RRR	RRRWRRLTV	HLA-B*14:02	236	19	54
LMP2A	TVC	TVCGGIMFL	HLA-A*02:01	243	3	5
LMP2A	LIV	LIVDAVLQL	HLA-A*02	257	2	3
LMP2A	GLG	GLGTLGAAL	HLA-A*02:01	293	2	4
LMP2A	LLW	LLWTLVVLL	HLA-A*02:01	329	18	32
LMP2A	SSC	SSCSSCPLSK	HLA-A*11:01	340	9	36
LMP2A	CPL	CPLSKILL	HLA-B*08	345	2	7
LMP2A	ILL	ILLARLFLY	HLA-A*0201	351	4	5
LMP2A	FLY	FLYALALLL	HLA-A*0201	356	31	62
LMP2A	GGS	GGSILQTNFKSLSSTEF	HLA-DPB1	372	2	16
LMP2A	TYG	TYGPVFMCL	HLA-A*24:02	419	22	33
LMP2A	CLG	CLGGLLTMV	HLA-A*02:01	426	99	230
LMP2A	LLS	LLSAWILTA	HLA-A*02:03	447	2	3
LMP2A	LTA	LTAGFLIFL	HLA-A*02:06	453	5	8
EBNA1	RPQ	RPQKRPSCI	HLA-B*35:01	72	3	5
EBNA1	HPV	HPVGEADYFEY	HLA-B*35:01	411	37	124
EBNA1	VPP	VPPGAIEQGPTDDPGEGPST	HLA-DR1, DR3, DQ2, DQ5	433	2	2
EBNA1	DGG	DGGRRKKGGWFGKHR	n.d.	459	2	4
EBNA1	NKP	NPKFENIAEGLRALL	HLA-DR11	479	4	8
EBNA1	AEG	AEGLRALLARSHVER	HLA-DQ7	486	2	4
EBNA1	LRA	LRALLARSHVERTTD	HLA-DQ3, DQ7, DQB1	489	2	2
EBNA1	FVY	FVYGGSKTSL	HLA-C*03:04	512	3	4
EBNA1	VYG	VYGGSKTSLYNLRRGTALAI	HLA-DR11, DR13, DQ3, DQ6	513	3	4
EBNA1	YNL	YNLRRGTAL	HLA-B*0801	522	3	6
EBNA1	IPQ	IPQCRLTPL	HLA-B*0702	532	6	8

EBNA1	APG	APGPGPQPGPLRESIVCYFM	HLA-DR1, DR3, DQ2, DQ5	548	2	2
EBNA1	FMV	FMVFLQTHI	HLA-A*0201	566	4	8
EBNA1	LQT	LQTHIFAEV	HLA-A*02:06	570	2	4
EBNA1	VLK	VLKDAIKDL	HLA-A*0201	578	4	4
EBNA1	PTC	PTCNIKATVCSFDDGVDLPP	HLA-DR2	593	2	3
EBNA3A	QAK	QAKWRLQTL	HLA-A*02:01 HLA-B*08:01	158	27	40
EBNA3A	AYS	AYSSWMYSY	HLA-A*24:02 HLA-A*29:02 HLA-C*07:02	176	5	5
EBNA3A	RYS	RYSIFFDYM	HLA-A*24:02	246	5	16
EBNA3A	AWN	AWNAGFLRGRAYGID	HLA-B*08	320	1	3
EBNA3A	FLR	FLRGRAYGL	HLA-B*08	325	89	259
EBNA3A	EDL	EDLPCIVSRGGPKVKRPPIF	HLA-DR15	364	2	4
EBNA3A	RPP	RPPIFIRRL	HLA-B*07	379	52	122
EBNA3A	LEK	LEKARGSTY	HLA-B*15	406	6	8
EBNA3A	HLA	HLAAQGMAY	HLA-B*46:01	450	4	6
EBNA3A	YPL	YPLHEQHGM	HLA-B*35	458	35	64
EBNA3A	VPA	VPAPAGPIV	HLA-B*07	502	6	8
EBNA3A	SVR	SVRDRLARL	HLA-A*02	596	15	31
EBNA3A	RLR	RLRAEAQVK	HLA-A*03:01	603	20	28
EBNA3A	GPW	GPWVPEQWMFQGAPPSQGTD	HLA-DR1	780	2	5

* Epitope data were collected from Immune Epitope Database (IEDB) and updated in July 2023.

Table S2 List of murine T-cell epitopes in LMP2A and EBNA1.

Antigen	Included in Trunc-EBNA1 or Trunc-LMP2A?	T cell epitopes	MHC restriction	Starting Position (aa)	Number of References*	Number of Assays*
EBNA1	Yes	VYGGSKTSL	H2-kd	512	1	2
EBNA1	Yes	EADYFEYHQEGGPDGE	H2-kd	415	1	2
LMP2A	Yes	AAALALLASLIL	H2-Kd	305	1	2
LMP2A	Yes	TYGPVFMCL	H2-Kd	419	2	2

* Epitope data were collected from Immune Epitope Database (IEDB) and updated in July 2023.

Table S3 The sequence of 5′-UTR and 3′-UTR

5′-UTR	GGGAAATAAGAGAGAAAAGAAGAGTAAGAAGAAATATAAGAGCCACC
3′-UTR	ACGCGTCTGGTACTGCATGCACGCAATGCTAGCTGCCCCTTTCCCGT CCTGGGTACCCCGAGTCTCCCCCGACCTCGGGTCCCAGGTATGCTC CCACCTCCACCTGCCCCACTCACCACCTCTGCTAGTTCCAGACACCT CCCAAGCACGCAGCAATGCAGCTCAAAACGCTTAGCCTAGCCACAC CCCCACGGGAAACAGCAGTGATTAACCTTTAGCAATAAACGAAAGTT TAACTAAGCTATACTAACCCCAGGGTTGGTCAATTTCGTGCCAGCCA CACC
